# Prediction of Room‐Temperature Superconductivity in Quasi‐Atomic H_2_‐Type Hydrides at High Pressure

**DOI:** 10.1002/advs.202405561

**Published:** 2024-07-21

**Authors:** Qiwen Jiang, Defang Duan, Hao Song, Zihan Zhang, Zihao Huo, Shuqing Jiang, Tian Cui, Yansun Yao

**Affiliations:** ^1^ Key Laboratory of Material Simulation Methods & Software of Ministry of Education and State Key Laboratory of Superhard Materials College of Physics Jilin University Changchun 130012 China; ^2^ Institute of High Pressure Physics School of Physical Science and Technology Ningbo University Ningbo 315211 China; ^3^ Synergetic Extreme Condition User Facility College of Physics Jilin University Changchun Jilin 130012 China; ^4^ Department of Physics and Engineering Physics University of Saskatchewan Saskatoon Saskatchewan S7N 5E2 Canada

**Keywords:** electron–phonon coupling, first‐principles, high pressure, hydride, superconductivity

## Abstract

Achieving superconductivity at room temperature (RT) is a holy grail in physics. Recent discoveries on high‐*T*_c_ superconductivity in binary hydrides H_3_S and LaH_10_ at high pressure have directed the search for RT superconductors to compress hydrides with conventional electron–phonon mechanisms. Here, an exceptional family of superhydrides is predicated under high pressures, *M*H_12_ (*M* = Mg, Sc, Zr, Hf, Lu), all exhibiting RT superconductivity with calculated *T*_c_ *s* ranging from 313 to 398 K. In contrast to H_3_S and LaH_10_, the hydrogen sublattice in *M*H_12_ is arranged as quasi‐atomic H_2_ units. This unique configuration is closely associated with high *T*_c_, attributed to the high electronic density of states derived from H_2_ antibonding states at the Fermi level and the strong electron–phonon coupling related to the bending vibration of H_2_ and H‐*M*‐H. Notably, MgH_12_ and ScH_12_ remain dynamically stable even at pressure below 100 GPa. The findings offer crucial insights into achieving RT superconductivity and pave the way for innovative directions in experimental research.

## Graphical abstract

A distinct family of superhydrides *M*H_12_ (*M* = Sc, Mg, Zr, Hf, Lu) with a novel quasi‐atomic H_2_ sublattice is predicted under high pressure. Based on first‐principles calculations, all *M*H_12_ compounds exhibit room‐temperature superconductivity, with calculated *T*_c_s ranging from 313 to 398 K. ScH_12_ and MgH_12_ are dynamically stable at pressures below 100 GPa.

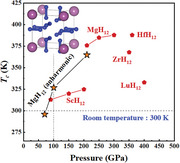

## 1 Introduction

It was predicted that solid hydrogen would become metallic in the atomic state under extreme compression.^[^ ^1^ ^]^ Theoretically, metallic hydrogen has all the ingredients needed for a room‐temperature (RT) superconductor, e.g., high‐frequency phonons, high density of states (DOS) at the Fermi level, and very strong electron–phonon coupling (EPC).^[^ ^2^ ^]^ However, the direct compression of solid hydrogen may require pressure over 500 GPa^[^ ^3^, ^4^, ^5^ ^]^ to reach a metallic state, which poses extreme difficulty in experiments. Alternatively, in compressed hydrides, the hydrogen species are ‘precompressed’^[^ ^6^ ^]^ by the metal elements, and therefore, the charge density sufficient for metallization can be achieved at less physical compression.^[^ ^7^, ^8^, ^9^, ^10^ ^]^ Currently, all experimentally synthesized high‐*T*_c_ hydrides necessitate high pressures above 150 GPa, but *T*_c_ below RT.^[^ ^11^, ^12^, ^13^, ^14^, ^15^, ^16^, ^17^, ^18^, ^19^ ^]^ The primary goal in physics and material science is to achieve RT superconductivity at pressures below 100 GPa, ultimately reaching ambient pressure conditions.

Diatomic H_2_ is a ubiquitous building block of solid hydrogen (e.g., phase I, II, III^[^ ^20^, ^21^, ^22^ ^]^) and of many hydrides as well,^[^ ^7^ ^]^ such as theoretically predicted SiH_4_(H_2_)_2_ and TeH_4_,^[^ ^23^, ^24^ ^]^ and experimentally synthesized BaH_12_,^[^ ^25^ ^]^ SrH_22_,^[^ ^26^ ^]^ HfH_14_,^[^ ^27^ ^]^ and SnH_12_,^[^ ^28^ ^]^ etc. These ‘molecular’ hydrides usually exhibit moderate superconductivity with a *T*_c_ below 120 K since the hydrogen electrons occupy bonding orbitals far below the Fermi level, resulting in a low DOS, even an insulating state. Breaking H_2_ molecular units is a prerequisite for achieving high‐*T*_c_ superconductivity.^[^ ^29^, ^30^, ^31^, ^32^ ^]^ To increase the *T*_c_ in hydrides, one needs to ‘free’ electrons from their locked positions, that is, to weaken the H_2_ units toward atomic hydrogen.^[^ ^30^ ^]^ This will allow the H electrons to occupy energy states closer to the Fermi level. Two prototypic hydrides consisting of atomic hydrogen sublattices, the cubic H_3_S^[^ ^11^, ^12^, ^33^, ^34^ ^]^ and cagelike LaH_10_,^[^ ^13^, ^14^, ^15^, ^30^, ^35^, ^36^ ^]^ were predicted following this principle and subsequently confirmed by experiments. These two hydrides have very high measured *T*_c_s in the 200–260 K range. Thus, the high‐*T*_c_ hydrides are recognized as being associated with weakened H_2_ units.

In the present work, we have predicted a novel group of dodecahydrides, *M*H_12_ (*M* = Mg, Sc, Zr, Hf, Lu), calculated to have *T*_c_ values exceeding RT. Different from previously reported atomic (H_3_S), cage (LaH_10_), and planar (HfH_10_^[^ ^37^ ^]^) forms, hydrogen atoms in *M*H_12_ are regularly arranged in quasi‐atomic peanut‐like H_2_ units. All dodecahydrides in this study are predicted to be RT superconductors with *T*_c_ up to 313–398 K estimated by solving Eliashberg equations. In particular, ScH_12_ and MgH_12_ are the first RT hydride superconductors hitherto predicted to exist at pressures below 100 GPa. Adding one H atom into the *M*H_12_, we obtained a class of *M*H_13_ containing quasi‐molecular H_2_ units, which also exhibit high *T*_c_s but are notably lower than their respective *M*H_12_ counterparts. Our research provides a promising approach for investigating phonon‐mediated RT superconductivity at lower pressures that meet or surpass the atomic metallic hydrogen.

## 2 Results and Discussion

**Figure 1:**
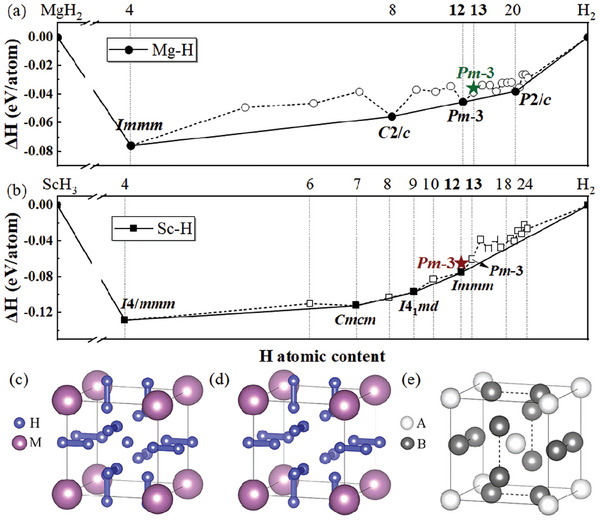
The thermodynamic convex hull diagram of a) Mg─H and b) Sc─H systems at 300 GPa with ZPE included. The red and green stars near the convex hull correspond to the *Pm* $\bar{3}$‐ScH_12_ and *Pm* $\bar{3}$‐MgH_13_ phases, respectively. Crystal structure of c) *M*H_13_, d) *M*H_12_, and e) the *A*15 type structure (AB_3_, *Pm* $\bar{3}$ *n*).

Recently reported high‐*T*_c_ hydrides mainly have the metal elements in the 2nd and 3rd groups of the periodic table.^[^ ^38^ ^]^ Mg and Sc represent the lightest elements among them (except for Be) and serve as the primary focus of our study. We performed variable composition structural searches for Mg─H and Sc─H systems at 200 and 300 GPa using AIRSS code.^[^ ^39^ ^]^ The convex hull diagram in **Figures** **1** and S1 (Supporting Information) illustrates the formation enthalpies for the optimal structures in all studied Mg─H and Sc─H stoichiometries. Zero‐point energy (ZPE) was included in the enthalpy calculation to account for the quantum effects. In this search, we successfully recovered the previously predicted structures from refs. [40, 41, 42] Moreover, two unique hydrides, *M*H_12_ and *M*H_13_ (*M* = Mg and Sc), with distinct *Pm* $\bar{3}$ symmetry, appeal to our attention. Both at 200 and 300 GPa, MgH_12_ and ScH_12_ lie on the convex hull, while ScH_13_ and MgH_13_ slightly surpass the hull by ≈8 meV/atom at 300 GPa. Due to synthesizing hydrides typically involving laser heating,^[^ ^18^, ^19^, ^43^, ^44^, ^45^ ^]^ we considered the temperature effect through quasi‐harmonic free energy calculations (Figure S2, Supporting Information). We found that the *Pm* $\bar{3}$ phase of MgH_12_ is more stable than the *R*‐3 phase at above 200 GPa and 1400 K, while the *Pm* $\bar{3}$ phase of ScH_12_ becomes more stable than the ground state *P*6_4_ and *Immm* phases at temperatures above 1700 K within the range of 200–400 GPa. These results suggest the synthesis potential of ScH_12_ and MgH_12_ within the pressure range of 200–300 GPa, alongside high temperatures.

To examine the dynamical stability of *M*H_12_ and *M*H_13_ (*M* = Mg and Sc), we calculated their phonon spectra at different pressures (Figure S6, Supporting Information). There are no imaginary phonon frequencies for MgH_12_ at 210 GPa and above, and for MgH_13_ at 300 GPa and above, which establish their dynamic stability ranges. ScH_12_ and ScH_13_ are dynamically stable down to a low pressure of 90 GPa. We extended the investigation to other isostructural *M*H_12_ and *M*H_13_ compounds involving other metal elements and identified Zr, Hf, and Lu, guided by criteria of similar Pauling electronegativity (≈1.3) and atomic radius (≈1.6 Å). Subsequent phonon dispersion and projected phonon DOS calculations confirm that these three metals' corresponding *M*H_12_ and *M*H_13_ are dynamically stable (Figures S6 and S7, Supporting Information). In comparison, *M*H_12_ and *M*H_13_ hydrides with Ca, Y, La, Ac, and Th are dynamically unstable up to 600 GPa due to large atomic radius or low electronegativity (Figures S8 and S9, Supporting Information).

In the $Pm\bar{3}-MH_{13}$, *M* atoms form a cubic lattice with a single H atom occupying the center. A pair of H_2_ units are perpendicular to and intersecting each face of the cube (Figure 1c). If the centered H atom is removed, the structure will turn into the $Pm\bar{3}-MH_{12}$ structure (Figure 1d). The structures of *M*H_12_ and *M*H_13_ are viewed as variants of the *A*15 type (AB_3_, *Pm* $\bar{3}$ *n*), with *M* and diatomic H_2_ being the two bases (see Figure 1e). The structural information is listed in Table S1 (Supporting Information). Interestingly, the *A*15 structure has been known to exist in conventional superconductors, such as Nb_3_Sn and Nb_3_Ge.^[^ ^46^ ^]^ There are also *A*15‐type superconductors in metal hydrides. For example, GaH_3_ and GeH_3_ have high *T*_c_ of 123 K at 120 GPa^[^ ^46^ ^]^ and 140 K at 180 GPa,^[^ ^47^ ^]^ respectively. Ternary hydride LiPH_6_^[^ ^48^ ^]^ also adopts the *A*15‐like configuration, which is predicted to be a high‐*T*_c_ superconductor (150–167 K at 200 GPa).

**Figure 2:**
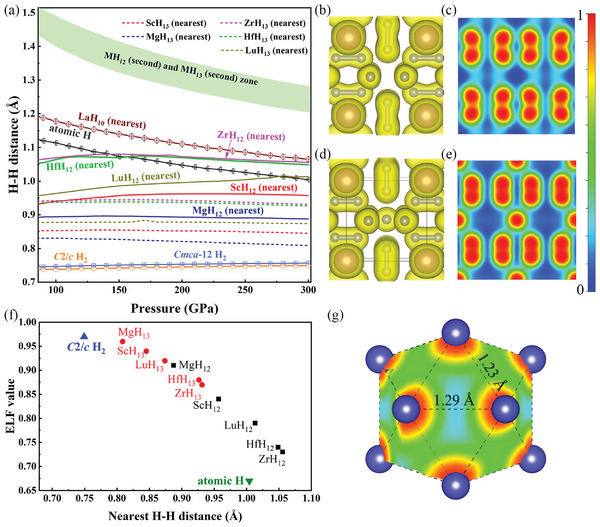
a) Distance between H atoms in *M*H_12_ (solid lines) and *M*H_13_ (dashed lines of the same color) as a function of pressure, compared with atomic and molecular hydrogen and LaH_10_. 3D ELF of b) ScH_12_ and d) ScH_13_ with isosurface value of 0.6. 2D ELF of c) ScH_12_ and e) ScH_13_ for the (0 0 2) plane at 300 GPa. f) The ELF value varies with the nearest H‐H distance of *M*H_12,13_, molecular H_2,_ and atomic H at 300 GPa. g) ELF cut plane of hydrogen icosahedron of ScH_12_ at 300 GPa.

The bonding characteristics of *M*H_12_ and *M*H_13_ are investigated using electron localization function (ELF), crystal orbital Hamiltonian population (COHP), and Bader charge analysis. Bader analysis (Table S2, Supporting Information) reveals significant charge transfer from the *M* atom to the H atoms, while each H_2_ pair receives more electrons in *M*H_12_ than in *M*H_13_. ScH_12,13_ serves as a typical example at 300 GPa. In ScH_12_, each H_2_ pair accepts 0.190 e^−^, while each H_2_ pair accepts 0.164 e^‒^ in ScH_13_. Since the electrons acquired by hydrogen must populate their antibonding states, this weakens the H─H bonds. In **Figure** **2**a, we compare the nearest and the second nearest H─H distances of *M*H_12_ and *M*H_13_ to those in atomic H (*I*4_1_/*amd*), molecular H_2_ solids (*C*2/*c* and *Cmca*‐12 phases), and LaH_10_. H─H contacts in both *M*H_12_ and *M*H_13_ are longer than the covalent bond length in molecular H_2_, as one would expect for weakened bonds. On average, shorter H─H bonds in *M*H_13_ than in *M*H_12_ are consistent with fewer electrons in the antibonding orbitals. Interestingly, similar to molecular H_2_, the H─H contact in *M*H_13_ does not change with pressure, indicating that the latter still contains molecular H_2_, albeit weaker in strength. We thus term the H_2_ units in *M*H_13_ as ‘quasi molecular’. On the other hand, the H─H bond length in *M*H_12_ keeps increasing at higher pressures, showing a pronounced tendency for H_2_ molecule dissociation, and reaches almost the same value as that in atomic hydrogen or LaH_10_ at 300 GPa. The important molecular feature of H_2_ molecular stretching vibrational modes near the frequency of 3000 cm^−1^ is absent in *M*H_12_ (see Figure S6, Supporting Information). Hence, it seems reasonable to refer to the H_2_ unit in *M*H_12_ as ‘quasi atomic’. The electron clouds surrounding quasi‐atomic H_2_ are shaped like peanuts, with a high ELF value of ≈0.84 for ScH_12_ (Figure 2b,c; Figures S10–S13, Supporting Information). Additionally, the ELF value between adjacent H_2_ units (perpendicular) at a distance of 1.23 Å is ≈0.45, exhibiting metallic interaction with free electrons connectivity (Figure 2g). As shown in Figure 2f, the H─H bonds in *M*H_12_ and *M*H_13_ display distinct electron localization characteristics, with the ELF values showing an approximately linear correlation with the H─H bond lengths. The calculated integrated crystalline orbital Hamiltonian population (ICOHP) values within the H_2_ units (Figures S14–S23, Supporting Information) are −3.23 eV in ScH_12_ and −4.91 eV in ScH_13_, indicating a much stronger bonding interaction in the latter. The ICOHP values between the H_2_ units (perpendicular) are −0.91 eV in ScH_12_ and −0.55 eV in ScH_13_. This manifests a weak inter‐molecular interaction in ScH_12,_ which causes the weakening of the H_2_ units.

**Figure 3:**
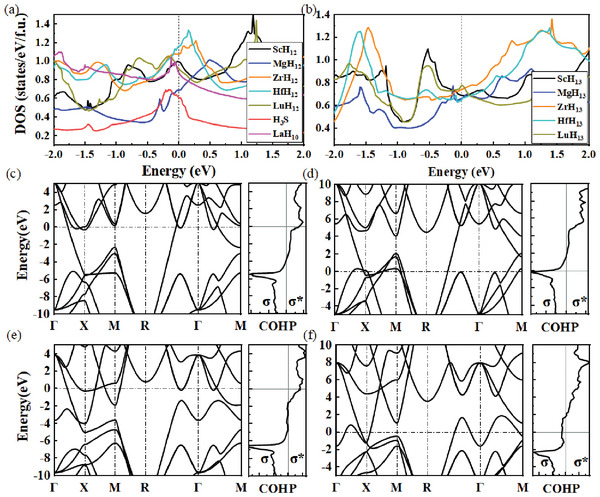
Total DOS of a) *M*H_12_ and b) *M*H_13_ compared to H_3_S and LaH_10_. Electronic band structures and COHP of H─H bonds for c) MgH_12_, d) Mg_0_H_12_, e) MgH_13_, and f) Mg_0_H_13_ at 300 GPa.

The electronic band structures and project DOS of *M*H_12_ and *M*H_13_ are shown in **Figure** **3** and Figures S24–S28 (Supporting Information). Given the uniqueness of magnesium superhydrides, we first discuss other *X*H_12, 13_ (*X* = Sc, Zr, Hf, Lu). A common feature displayed in these *X*H_12_ is the van Hove singularity with a large total DOS value at the Fermi level (Figure 3a), formed by strong hybridization of *d*‐orbital (*f*‐orbital) of metal atoms and the *s*‐orbital of H atoms (Figures S25–S28, Supporting Information). In contrast, the DOS in *X*H_13_ is notably uniform around the Fermi level (Figure 3b). The van Hove singularity plays an important role in enhancing the EPC and increasing *T*_c_ in H_3_S and LaH_10_. The comparison of *X*H_12_ to H_3_S and LaH_10_ at 300 GPa shows that *X*H_12_ has a significantly larger DOS at the Fermi level, with a trend of *X*H_12_ > LaH_10_ > *X*H_13_ > H_3_S (Figure 3a). High electronic DOS at the Fermi level, in particular those induced by hydrogen, sets a favorable condition for Cooper pairs, strong EPC, and superconductivity. The electronic structures of MgH_12_ and MgH_13_ are different from *X*H_12, 13_, characterized by nearly unoccupied *d*‐states of Mg in the valence bands, and H atoms significantly contribute to the total DOS (≈86%), as listed in Table S3 (Supporting Information). As shown in Figure 3c–f, the band structures of MgH_12_ and MgH_13_ highly resemble Mg_0_H_12_ and Mg_0_H_13_ (hypothetical structures with Mg removed from the hydrides, without relaxation), exhibiting a pure hydrogen character. The presence of Mg provides electrons to the H_2_ units, which elevate the Fermi level from the low‐energy bonding states (σ) to the antibonding states (σ^*^), maximizing the electronic states of H at the Fermi level. We observe interlocking electron pockets near the X point, especially in MgH_12_, where the nearly degenerate *A_g_* and *A_u_* bands at the X point and the hole pocket at the Γ point, create a fish‐tail‐shaped Fermi surface along the Γ‐X direction (Figure S29, Supporting Information). The subsequent peak of the nesting function ξ(Q) in that direction indicates strong Fermi surface nesting, which is advantageous for EPC (Figure S30, Supporting Information).

**Figure 4:**
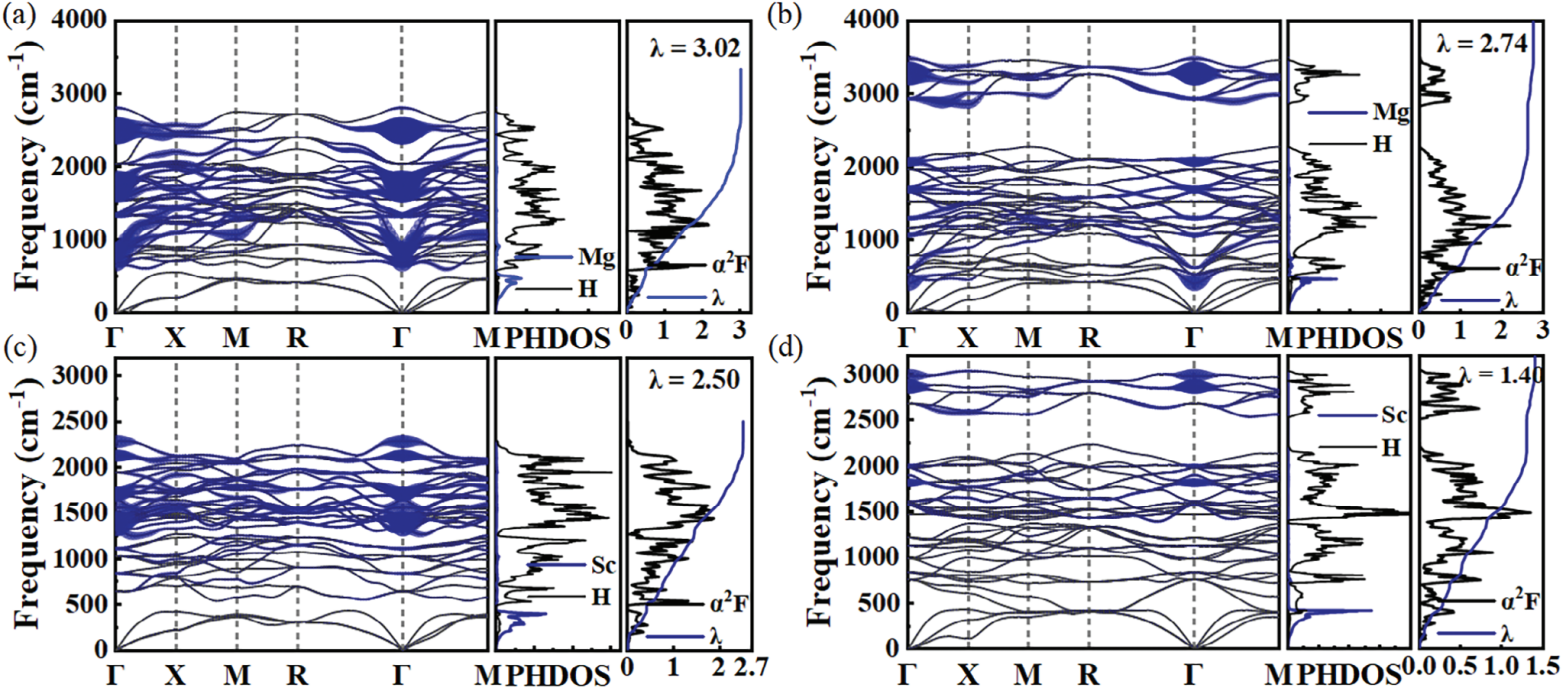
Phonon band structure (left), PHDOS (middle), and Eliashberg spectral function *α* ^2^ *F*(*ω*) (right) for (a) MgH_12_, b) MgH_13_, c) ScH_12,_ and d) ScH_13_ at 300 GPa. The blue circles on the phonon dispersion curves indicate the phonon linewidth, with the circle's radius proportional to the strength of the electron–phonon coupling.

To examine the superconducting properties of *M*H_12,13_, we calculated the logarithmic average phonon frequency (*ω*_log_), EPC parameter (*λ*), and Eliashberg phonon spectral function [*α* ^2^ *F*(*ω*)], as shown in **Figure** **4**. It should be noted that the vibrational bands of hydrogen in MgH_12_ and ScH_12_ hydrides are mixed and dispersive, with all frequencies below 2800 cm^−1^ at 300 GPa, consistent with the disappearance of molecular H_2_. For MgH_13_ and ScH_13_, the high‐frequency vibrations still form an isolated non‐dispersive block, peaking at ≈3000 cm^−1^. This frequency aligns with the stretching vibrations of quasi‐molecular H_2_, yet their contribution to the EPC is minor (< 10%). For pure molecular hydrogen *C*2/*c* and *Cmca*‐12 phases,^[^ ^49^ ^]^ the highest vibrational frequency is ≈4000 cm^−1^ at 250 GPa. The lower hydride vibrational frequencies indicate weaker H─H bonds induced by the interaction between H and metal atoms. Compared to *M*H_13_, the calculated *λ* values for all *M*H_12_ compounds within their dynamically stable pressure range are greater than or equal to 2.5, suggesting a very strong EPC. The remarkable λ values are mainly associated with mixed stretching, rocking, and scissoring vibrations of the H_2_ units within the mid‐frequency range, accompanied by the bending vibrations of H─Sc─H (Figure S31, Supporting Information). Using typical Coulomb pseudopotential *μ* ^*^ = 0.1–0.13, applying the Eliashberg equations yields a very high *T*_c_ value ranging 304–325 K for ScH_12_ with λ = 2.50 at 300 GPa (Table S4 and Figure S32, Supporting Information). In the case of ScH_13_, the highest *T*_c_ is calculated to be 185 K (*μ* ^*^ = 0.1) with λ = 1.40, much lower than that of ScH_12_. MgH_12_ and MgH_13_ exhibit RT superconductivity, with *T*_c_ values of 366–388 and 303–324 K at 300 GPa, respectively. *M*H_12_ (*M* = Zr, Hf, and Lu) are also calculated to have RT *T*_c_ between 333 and 388 K, and *M*H_13_ hosts high *T*_c_ with 160–200 K (*μ* ^*^ = 0.10).

**Figure 5:**
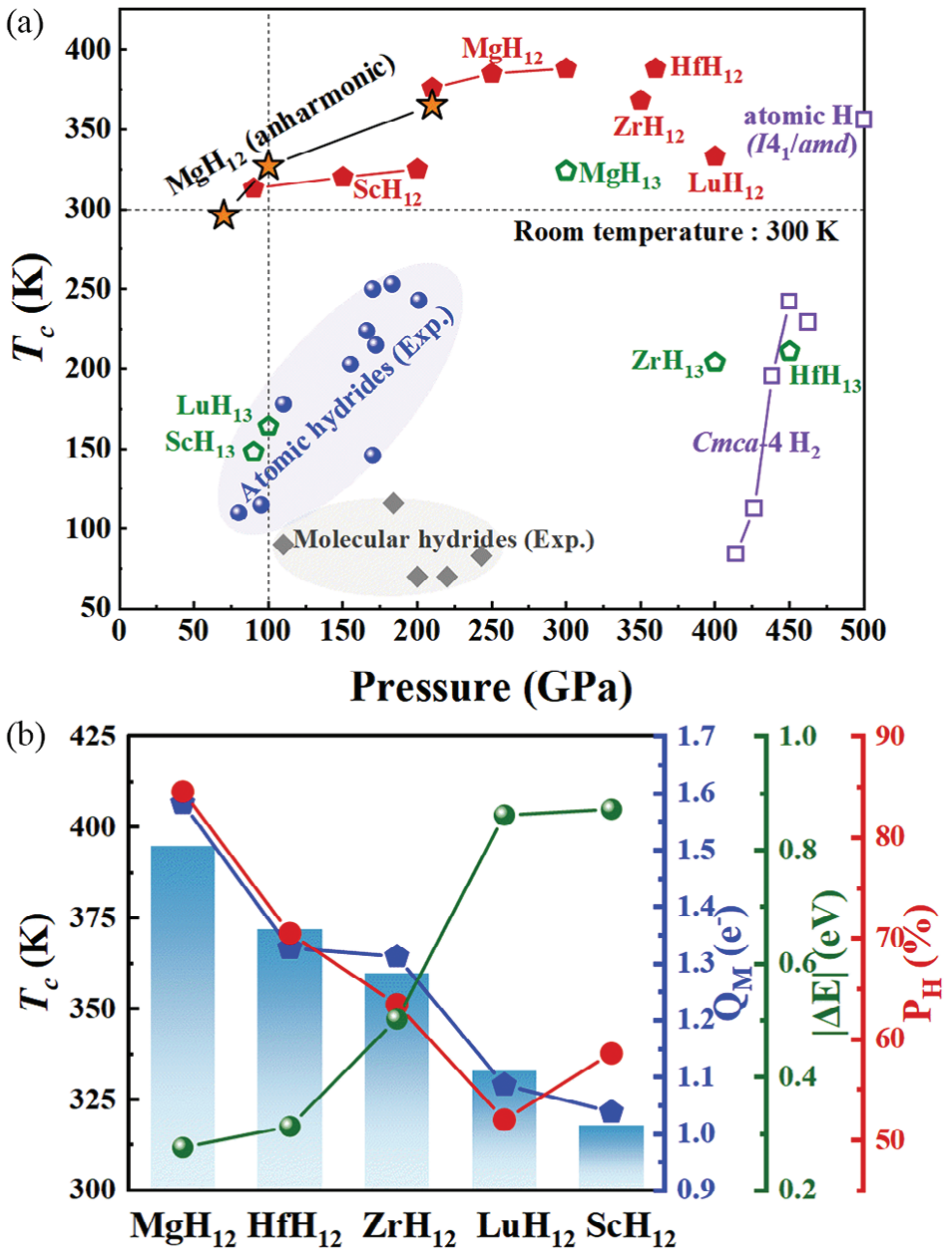
a) Calculated *T*_c_ of *M*H_12_ and *M*H_13_ by numerically solving Eliashberg equations at different pressures with *μ* ^*^ = 0.10. The blue circles and gray squares denote the well‐known hydrides from experiments.^[^ ^11^, ^12^, ^13^, ^14^, ^15^, ^16^, ^17^, ^18^, ^19^, ^27^, ^28^, ^44^, ^45^, ^54^, ^55^, ^56^, ^57^, ^58^, ^59^, ^60^ ^]^ Superconductivity in molecular hydrogen (*Cmca*‐4) and atomic hydrogen (*I*4_1_/*amd*) is documented in refs. [50, 61] b) Calculated at 400 GPa for *M*H_12_, the *T*_c_, the electrons transferred from *M* to hydrogen atoms (Q*_M_*), the percentage of the H electronic DOS at the Fermi level (P_H_), and the distance between the H‐H antibonding state and the Fermi level at the X point (|ΔE|).

As shown in **Figure** **5**a, the superhydrides *M*H_12_ investigated in this study exhibit remarkably high *T*_c_ values comparable to atomic metallic hydrogen (≈356 K at 500 GPa),^[^ ^50^ ^]^ particularly with substantially reduced stability pressures in Sc and Mg. This highlights the potential of *M*H_12_ superhydrides as promising candidates for RT superconductors. Strikingly, the superconductivity of ScH_12_ shows weak dependence on pressure, maintaining RT superconductivity down to a significantly lower pressure of 90 GPa when compared to other compressed hydrides (e.g., YH_10_ and Li_2_MgH_16_ at 250 GPa).

Given the light mass of hydrogen, anharmonicity is crucial for superhydrides, especially those with strong EPC.^[^ ^51^, ^52^ ^]^ Due to the substantial computational cost, we take MgH_12_ as an example and examine this effect using the stochastic self‐consistent harmonic approximation.^[^ ^53^ ^]^ As shown in Figure S33 (Supporting Information), the anharmonic correction hardens phonon modes near the Γ‐point, eliminating instability above 70 GPa. At 210 GPa, the anharmonicity weakens the EPC parameter while enhancing *ω*_log_, resulting *T*_c_ slightly decreasing to 345–365 K (Table S5, Supporting Information), maintaining above RT. At 70 GPa, the anharmonic *T*_c_ value is 281–296 K, still close to RT.

As discussed above, the intriguing RT superconductivity of *M*H_12_ is related to the transferred electrons from metal *M* to H atoms (*Q_M_*), the percentage of H electronic DOS (*P*_H_), and the energy difference (|Δ*E*|) between the flat band at the X point and the Fermi level (Figure S34, Supporting Information). To gain further insights, we plotted the relationship between these parameters and *T*_c_, as shown in Figure 5b. From Sc to Lu, Zr, Hf, and finally to Mg, there is an increasing electron transfer from metals to hydrogen, elevating the Fermi energy and leading to a decrease in |Δ*E*|. This brings the antibonding orbitals of hydrogen corresponding to the flat band closer to the Fermi level, maximizing the DOS contribution of hydrogen and promoting the formation of Cooper pairs. Consequently, the EPC constant for the optical modes derived from hydrogen at the X point gradually strengthens (Figure S35, Supporting Information), increasing *T*_c_.

In previous research, it was thought that hydrides containing H_2_ or H_3_ molecular units are unfavorable for superconductivity. However, this study found a particular case, MgH_13,_ with quasi‐molecular H_2_ units exhibiting RT superconductivity. As shown in Figure S36 (Supporting Information), the H─H bond length does not display a singular linear relationship with *T*_c_. Hence, inferring *T*_c_ based solely on this criterion appears to be rudimentary. We further performed a statistical analysis of the relationship between *T*_c_ and the product of *P*_H_ and the DOS per hydrogen atom (DOS_per H_), and the results show a positive correlation (Figure S37, Supporting Information). It is necessary to calculate the DOS of hydrogen at the Fermi level to predict the possibility of hydride superconductivity.^[^ ^62^ ^]^ The pure hydrogen framework of H_12_ offers a plethora of unoccupied energy levels. In certain s/p block metal hydrides like Mg, extra electrons support the prominent DOS associated with the dominant antibonding states of H_2_, albeit with a minor inclination toward molecular dissociation.

## 3 Conclusion

In summary, an extensive structure search combined with ab initio calculations reveals a unique superhydride *M*H_12_ (*M* = Mg, Sc, Zr, Hf, Lu) under high pressure. In this structure, hydrogen atoms are arranged in quasi‐atomic peanut‐like H_2_ units, which differs significantly from the hydrogen configurations in known high‐*T*_c_ hydrides H_3_S and LaH_10_. *M*H_12_ is calculated to have a superconducting critical temperature *T*_c_ similar to or superior to metallic hydrogen solids, exhibiting superconductivity at the highest temperature among all known/predicted binary hydrides. In *M*H_13_, hydrogen atoms form quasi‐molecular H_2_ units, which are also predicted to possess high *T*_c_. The intriguing family of RT superconducting hydrides we have predicted is poised to stimulate future high‐pressure experimental investigations, potentially enabling synthesis within diamond anvil cells through laser heating.

## Conflict of Interest

The authors declare no conflict of interest.

## Supporting information

Supporting Information

## Data Availability

The data that support the findings of this study are available in the supplementary material of this article.
