# Supplementary material for: Prediction of Room‐Temperature Superconductivity in Quasi‐Atomic H2‐Type Hydrides at High Pressure
Source: Adv Sci (Weinh). 2024 Jul 21;11(35):2405561. doi: 10.1002/advs.202405561 (PMC11425200; doi:10.1002/advs.202405561)
Supplement: Supplementary file 1 — Supporting Information [file ADVS-11-2405561-s001.pdf]

Supporting Information

Prediction of Room-Temperature Superconductivity in Quasi-atomic H_2-Type Hydrides at High Pressure

Qiwen Jiang[1], Defang Duan[1,*], Hao Song[2], Zihan Zhang[1], Zihao Huo[1], Shuqing Jiang[3,1], Tian Cui[2,1,*], Yansun Yao[4]

[1]*Key Laboratory of Material Simulation Methods & Software of Ministry of Education and State Key Laboratory of Superhard Materials, College of Physics, Jilin University, Changchun 130012, China*

[2]*Institute of High Pressure Physics, School of Physical Science and Technology, Ningbo University, Ningbo, 315211, China*

[3]*Synergetic Extreme Condition User Facility, College of Physics, Jilin University, Changchun, Jilin 130012, China*

[4]*Department of Physics and Engineering Physics, University of Saskatchewan, Saskatoon, Saskatchewan S7N 5E2, Canada*

Contents

Computational details 3
Thermodynamic stability of *M*-H systems 6
Dynamic stability and superconductivity of MH_{12} and MH_{13} 9
Structural information 13
Bader charges analysis 14
Bond analysis 15
Electronic properties analysis 22
Phonon vibration modes 25
Superconductive parameters 27
Anharmonic phonon correction and superconductivity 29
Superconducting mechanism analysis 30
References 33

Computational details

Variable composition searches of the high-pressure ground-state structures were conducted using *ab initio* random structure searching (AIRSS) [1,2] and Cambridge serial total energy package (CASTEP) [3] code based on density functional theory (DFT) [4,5]. A cell volume and shape are randomly selected within a reasonable range, and atoms are added at random positions to achieve the desired stoichiometry using AIRSS code. All predicted structures undergo relaxation using the CASTEP code until the forces on the atoms become negligible and the pressure reaches the required value. For the searches in Mg-H and Sc-H systems, we used generalized gradient approximation with the Perdew-Burke-Ernzerhof (GGA-PBE) for the exchange-correlation functional, a plane wave cutoff energy of 300 eV and a Monkhorst-Pack [6] Brillouin zone sampling grid of spacing $2\pi \times 0.07$ Å^{-1}. The stoichiometries MgH_n and ScH_n (n=3-13) were simulated using 1-4 formula unit (f.u.) cells, while hydrogen-rich MgH_n and ScH_n (n=14-24) were simulated using 1-2 f.u. cells. The most favorable structures within 50 meV/atom from the hull were relaxed at a higher level of accuracy with a cutoff of 700 eV and a k-point grid spacing of $2\pi \times 0.03$ Å^{-1}. To search for structures of the Sc-H system, ultra-soft potentials were used, while on-the-fly (OTF) generation of ultrasoft potentials were employed for the structure searching of Mg-H, Zr-H, Hf-H, and Lu-H systems. The number of predicted structures at stoichiometries $M\mathrm{H}_{12}$ and $M\mathrm{H}_{13}$ (M = Mg, Sc, Zr, Hf, Lu) was more than 1000.

The electronic properties were performed in the framework of DFT within the GGA-PBE method [7], as implemented in the Vienna *Ab-initio* Simulation Package (VASP) code [8]. The projector-augmented wave [9] approach was adopted to describe ion-electron interactions, where $1s^1$, $2p^6 3s^2$, $3s^2 3p^6 4s^1 3d^2$, $4s^2 4p^6 4d^2 5s^2$, $5s^2 5p^6 5d^2 6s^2$, and $4f^{14} 5s^2 5p^6 5d^1 6s^2$ are considered as valence electrons for H, Mg, Sc, Zr, Hf, and Lu atoms, respectively. A plane-wave basis set with an energy cutoff of 800 eV was used to get well-converged total energies. Electron localization function (ELF) [10], with values ranging from 0 to 1, was calculated to describe and visualize chemical bonds in multi-electron systems. The values of 0 and 1 represent complete delocalization and localization of the electrons, respectively. ELF =0.5 corresponds to perfect free-electron gas distribution. The Crystalline Orbital Hamiltonian Population (COHP) and Integrated Crystalline Orbital Hamiltonian Population (ICOHP) are calculated using the LOBSTER code [11], commonly used to distinguish between covalent and non-covalent bonds

in chemistry. For charge transfer between metal atoms and H atoms, we use Bader charge analysis [12].

Due to the non-negligible quantum effect caused by the extremely light mass of the H atom, the effect of zero-point energy (ZPE) on the calculation of formation enthalpies of predicted compounds is considered in the harmonic approximation by the formula:

$$E_{ZPE} = \frac{1}{2}\sum_{q,j} \hbar\omega_{\mathrm{j}}\,(q),$$

where j indicates a phonon branch at wave vector q; $\omega_j(q)$ is the frequency at wave vector q.

For specific structures, we further investigated the temperature effects using the quasiharmonic approximation (QHA) that introduces volume dependence of phonon frequencies as a part of the anharmonic effect [13]. Gibbs free energy (G) is defined at a constant temperature (T) and pressure (p) by the formula:

$$G(T,p) = \min[U(V) + F_{phonon}(T;V) + pV],$$

where U is the internal lattice energy, F_{phonon} is the phonon (Helmholtz) free energy, and V is the volume. Changing the volume to find the unique minimum in the square brackets and get G. In our calculation, 5-10 volume points were taken into account. The harmonic approximation and the QHA are performed in the PHONOPY code [14]. For more detailed explanations, refer to the website: http://phonopy.github.io/phonopy/qha.html#phonopy-qha.

Phonon and electron-phonon coupling (EPC) calculations were performed using the QUANTUM ESPRESSO code [15]. Ultrasoft pseudopotentials [16] were employed for MgH_n, ScH_n, ZrH_n, and HfH_n, with a kinetic energy cutoff of 80 Ry. In the case of LuH_n, the lanthanide hydrides, we opted for the PAW pseudopotentials, taking into account the additional valence electrons of Lu ($4f^{14}5s^{2}5p^{6}5d^{1}6s^{2}$), and applied a kinetic energy cutoff of 100 Ry. The k-points and q-points meshes in the first Brillouin zone are 24 × 24 × 24 and 6 × 6 × 6 for MH$_{12}$ and MH$_{13}$ (M = Mg, Sc, Zr, Hf, Lu). T_c has been estimated through the Allen-Dynes-modified McMillan equation with correction factors [17] and Eliashberg equations [18].

The anharmonicity of MgH_{12} was determined through the variational stochastic self-consistent harmonic approximation (SSCHA) method [19,20]. The SSCHA calculations were performed using a 2 × 2 × 2 supercell containing 104 atoms at 0 K, yielding dynamical matrices on a commensurate 2 × 2 × 2 q-point grid in the Brillouin zone. A 70 Ry energy cutoff and a 5 × 5 × 5 Monkhorst−Pack k-point mesh for Brillouin zone integrations were sufficient in the supercell to converge the SSCHA gradient.

The total energies, forces, and stress tensors for the individuals are obtained from DFT calculations. At the end of a minimization run, a new population with a higher number of individuals N is generated from the minimized trial density matrix until convergence. We set two stopping criteria for the minimization loops: a Kong−Liu ratio for the effective sample size of 0.6 and a ratio of $<10^{-7}$ between the free energy gradient concerning the auxiliary dynamical matrix and its stochastic error. In calculations based on DFT, we increased the N up to 800 individuals. The total forces in the last population are well below 10^{-6} meV/Å. The physical phonon frequency differences between the last two populations are below 1 meV. The difference between the harmonic and anharmonic dynamical matrices in the $2 \times 2 \times 2$ q-point grid was interpolated to a $6 \times 6 \times 6$ grid. By adding the harmonic $6 \times 6 \times 6$ grid dynamical matrices to the result, the anharmonic $6 \times 6 \times 6$ q-grid dynamical matrices were obtained. Subsequently, a $24 \times 24 \times 24$ k-point grid and a $6 \times 6 \times 6$ q-point grid were used for the anharmonic EPC calculation.

Thermodynamic stability of *M*-H systems

The thermodynamic stability of MH_{12} and MH_{13} (*M*= Zr, Hf, and Lu) is determined in their corresponding convex hulls (Figures S2-S4) established by structures of all *M*-H stoichiometries, which are obtained in extensive structure search. With the ZPE accounted at 300 GPa, HfH_{12} are very close to the convex hull (4 meV/atom), and ZrH_{12} and LuH_{12} are slightly higher above (14-15 meV/atom). These metastable structures are still energetically favorable with respect to the starting material ($MH_3 + H_2$) and likely to be synthesized under high-pressure and high-temperature conditions.

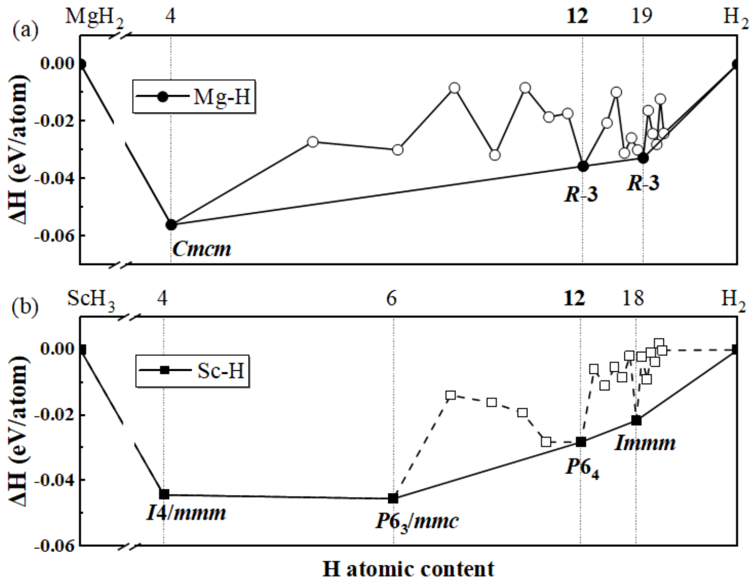

Figure S1. The thermodynamic convex hull diagram of (a) Mg-H and (b) Sc-H systems at 200 GPa with ZPE included.

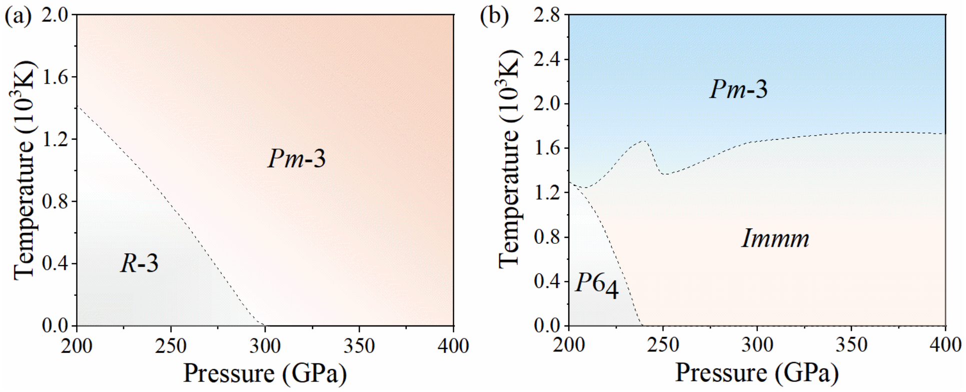

Figure S2. Temperature versus pressure phase diagram of (a) MgH_{12} and (b) ScH_{12}.

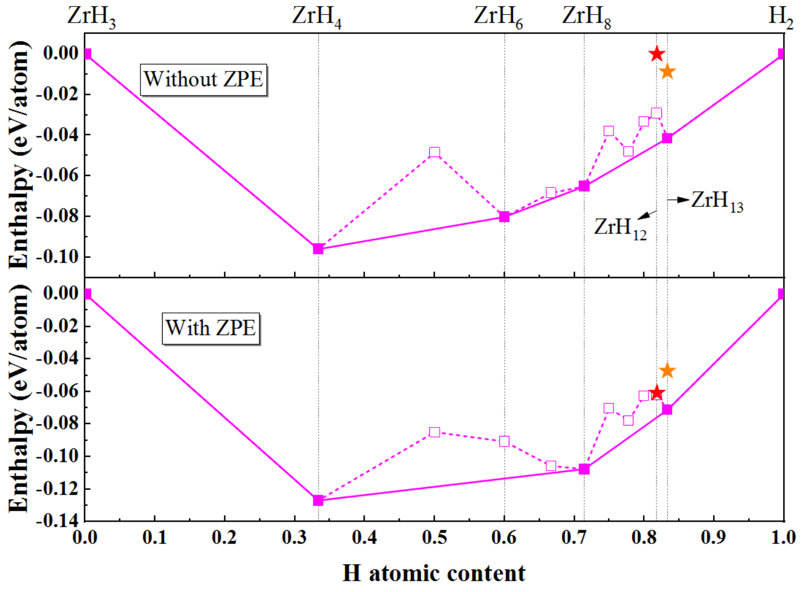

Figure S3. The thermodynamic convex hull diagram of Zr-H with respect to ZrH_3 and H_2 at 300 GPa, without (top) and with (bottom) ZPE. The red and orange stars near the convex hull represent the $Pm\bar{3}$ phase of ZrH_{12} and ZrH_{13}, respectively. Considering ZPE, the red and orange stars are 15 meV/atom and 24 meV/atom above the convex hull.

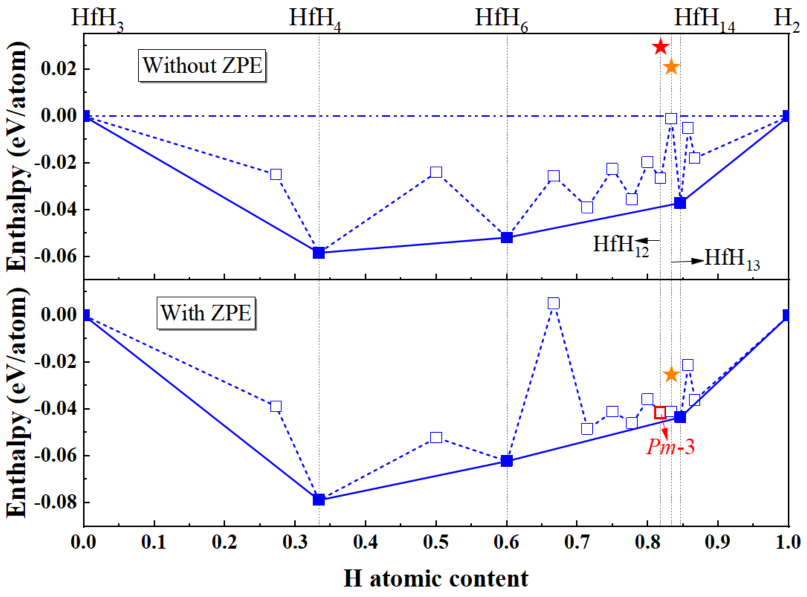

Figure S4. The thermodynamic convex hull diagram of Hf-H with respect to HfH_3 and H_2 at 300 GPa, without (top) and with (bottom) ZPE. The red star or hollow square represents the $Pm\bar{3}$-HfH_{12} phase, and the orange star represents the $Pm\bar{3}$-HfH_{13} phase. Considering ZPE, the red open square and orange star are 4 meV/atom and 19 meV/atom above the convex hull.

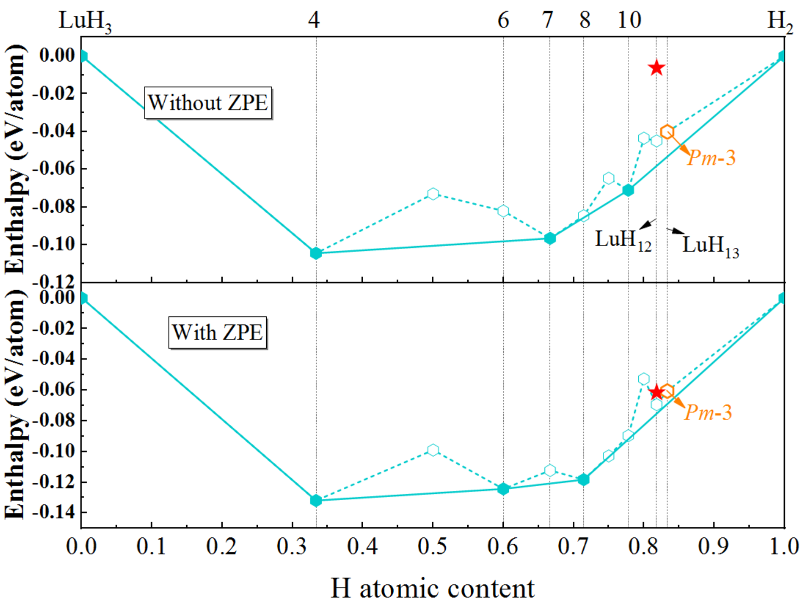

Figure S5. The thermodynamic convex hull diagram of Lu-H with respect to LuH_3 and H_2 at 300 GPa, without (top) and with (bottom) ZPE. The red star and the orange hollow pentagon near the convex hull represent the $Pm\bar{3}$ phase of LuH_{12} and LuH_{13}, respectively. Considering ZPE, the red star and orange open pentagon are 14 meV/atom and 8 meV/atom above the convex hull.

Dynamic stability and superconductivity of MH_{12} and MH_{13}

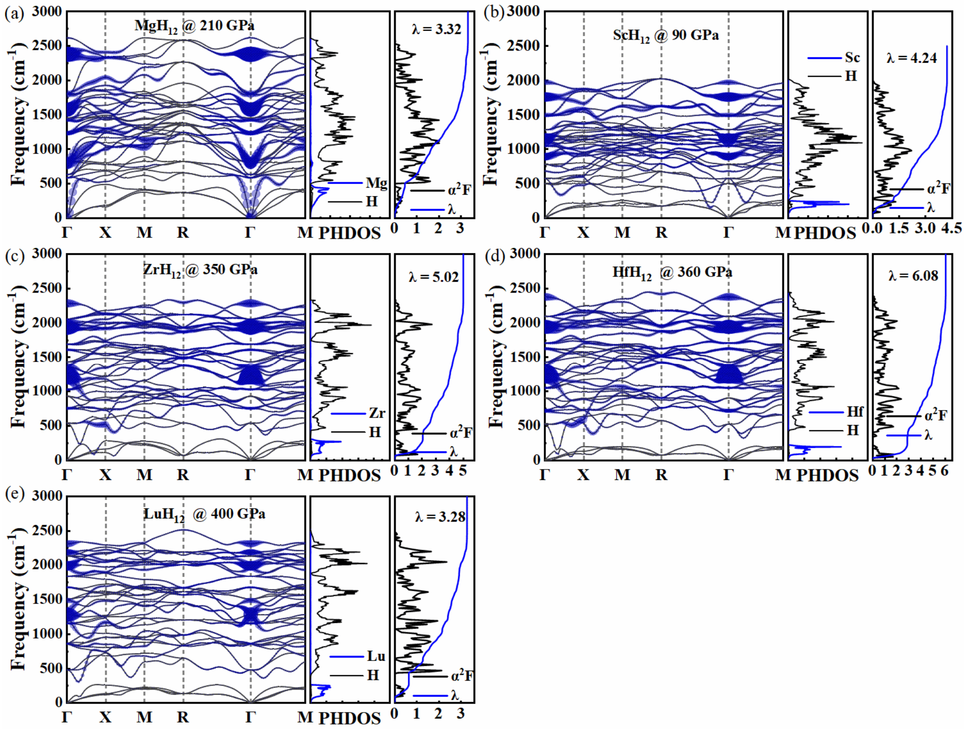

Figure S6. Phonon properties and Eliashberg spectral function of MH_{12} (M = Mg, Sc, Zr, Hf and Lu) are presented at the pressures where they start to become dynamically stable. Phonon band structure (left), PHDOS (middle), and Eliashberg spectral function $\alpha^2F(\omega)$ (right) for (a) MgH_{12} at 210 GPa, (b) ScH_{12} at 90 GPa, (c) ZrH_{12} at 350 GPa (d) HfH_{12} at 360 GPa and (e) LuH_{12} at 400 GPa. The size of the blue circle indicates the contribution to the phonon line width.

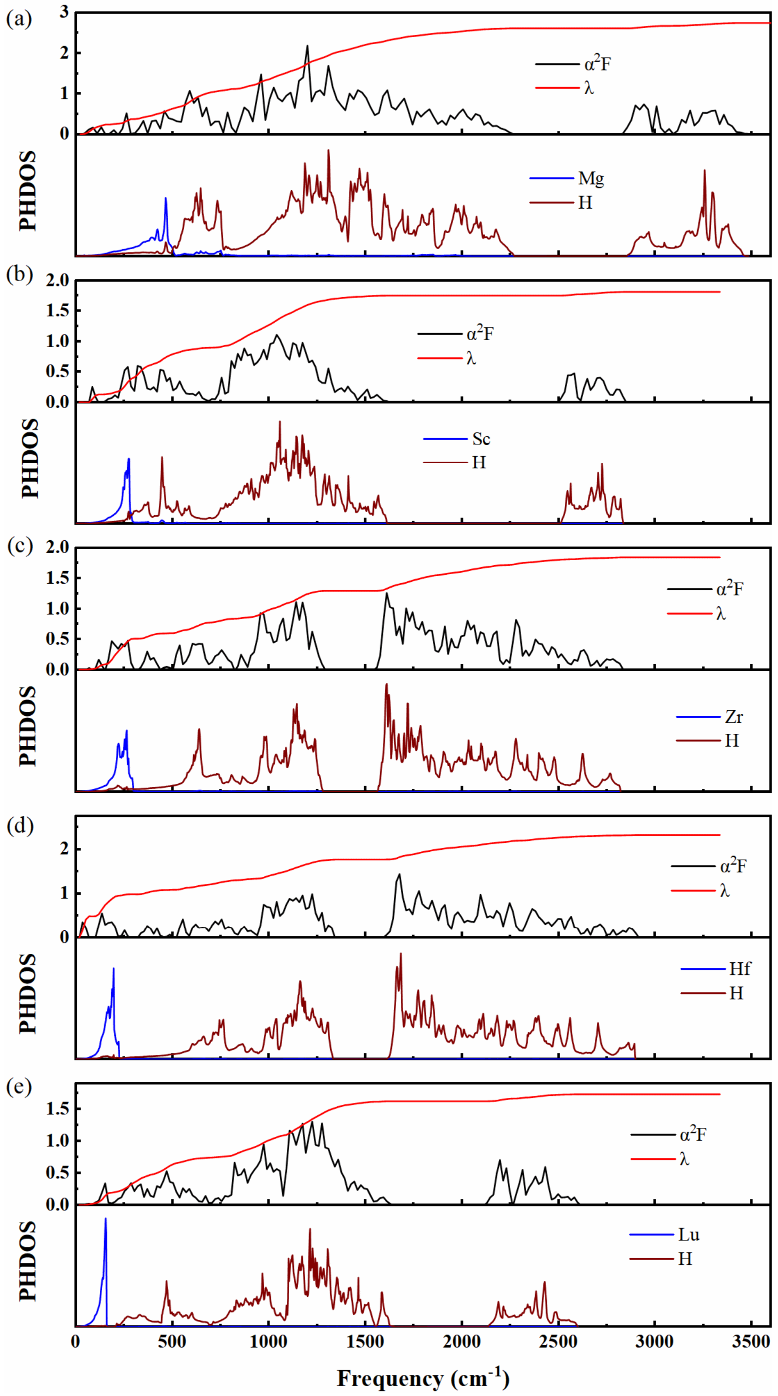

Figure S7. Calculated projected phonon density of states (lower panels), Eliashberg spectral function $\alpha^2F(\omega)$ and its integral λ (upper panels) of MH_{13} (M = Mg, Sc, Zr, Hf and Lu) are presented at the pressures where they start to become dynamically stable. (a) ScH_{13} at 90 GPa, (b) MgH_{13} at 300 GPa, (c) ZrH_{13} at 400 GPa, (d) HfH_{13} at 450 GPa, and (e) LuH_{13} at 100 GPa.

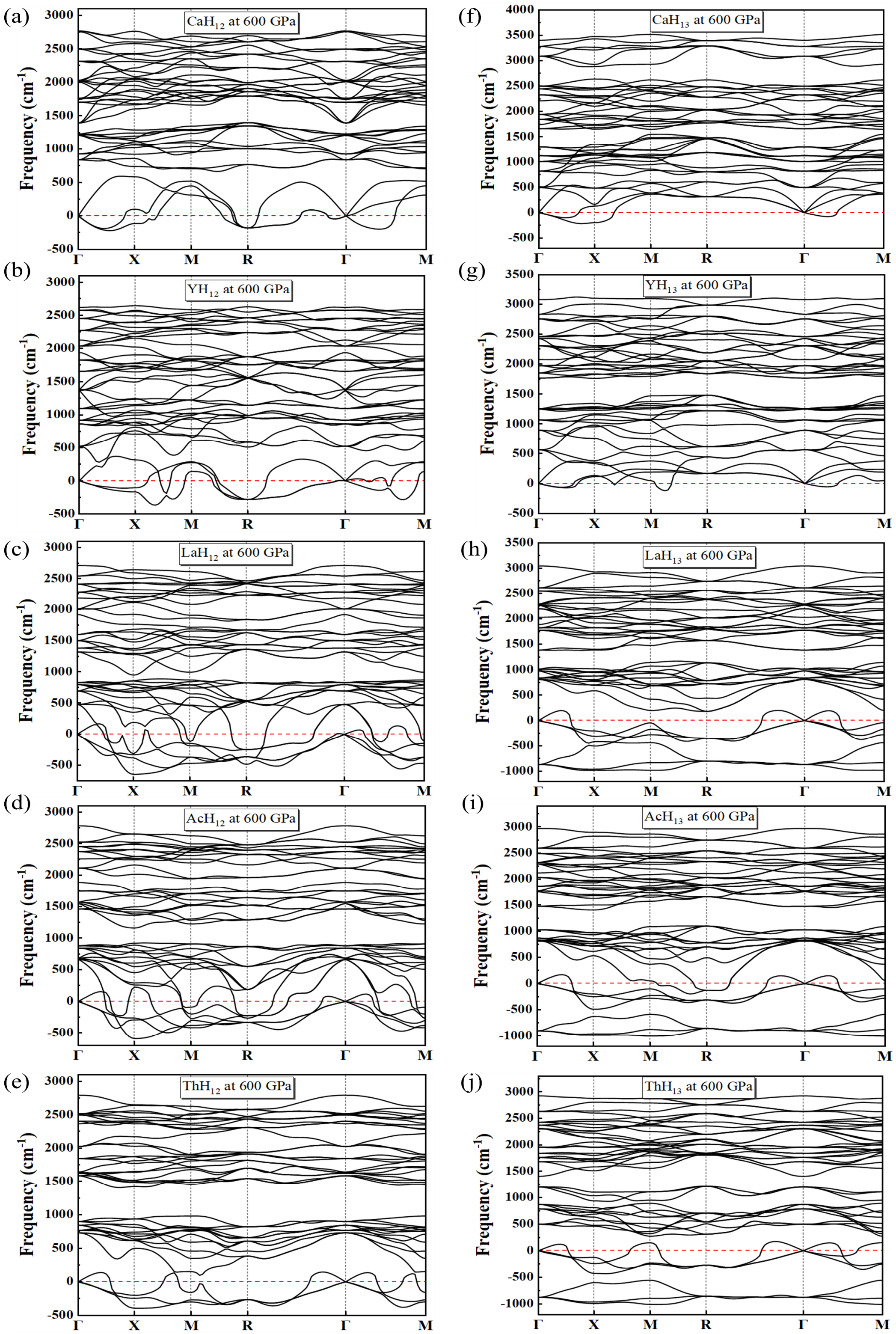

Figure S8. Phonon dispersion curves for (a) CaH_{12}, (b) YH_{12}, (c) LaH_{12}, (d) AcH_{12}, (e) ThH_{12}, (f) CaH_{13}, (g) YH_{13}, (h) LaH_{13}, (i) AcH_{13}, and (j) ThH_{13} at 600 GPa.

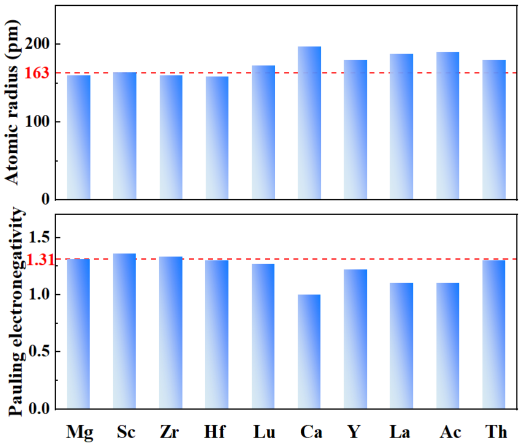

Figure S9. Atomic radius and Pauli electronegativity for the *M* elements in MH_{12} compared to those in Ca, Y, La, Ac, Th.

Structural information

Table S1. Structural information of predicted hydrides at 300 GPa.

Compound	Space group	Lattice Parameters (Å, °)	Atoms	Atomic coordinates (fractional)		
MgH_{12}	$Pm\bar{3}$	$a = b = c = 2.8620$	H (12j)	0.22090	-0.00000	0.34495
		$\alpha = \gamma = \beta = 90$	Mg (1b)	0.50000	0.50000	0.50000
MgH_{13}	$Pm\bar{3}$	a=b=c = 2.9239	H (12k)	0.50000	0.27353	0.86171
		α=γ=β=90	H (1b)	0.50000	0.50000	0.50000
			Mg (1a)	0.00000	0.00000	0.00000
ScH_{12}	$Pm\bar{3}$	$a = b = c = 2.9326$	H (12j)	0.21990	0.00000	0.33681
		$\alpha = \gamma = \beta = 90$	Sc (1b)	0.50000	0.50000	0.50000
ScH_{12}	$P6_4$	$a = b = 4.0784$,	H (6c)	0.043220	0.182880	0.896810
		c = 5.3683	H (6c)	0.461080	0.801670	0.443390
		$\alpha = \beta = 90$, $\gamma = 120$	H (6c)	0.961580	0.291880	0.064130
			H (6c)	0.650670	0.445070	0.267290
			H (6c)	0.043990	0.210260	0.744050
			H (6c)	0.332180	0.040230	0.885790
			Sc (3b)	0.500000	0.500000	0.828330
ScH_{13}	$Pm\bar{3}$	$a = b = c = 2.9835$	H (12k)	0.50000	0.85848	0.72593
		$\alpha = \gamma = \beta = 90$	H (1b)	0.50000	0.50000	0.50000
			Sc (1a)	0.00000	0.00000	0.00000
HfH_{12}	$Pm\bar{3}$	$a = b = c = 3.0025$	H (12j)	0.21253	-0.00000	0.32533
		$\alpha = \gamma = \beta = 90$	Hf (1b)	0.50000	0.50000	0.50000
HfH_{13}	$Pm\bar{3}$	$a = b = c = 3.0541$	H (12k)	0.50000	0.28041	0.84832
		$\alpha = \gamma = \beta = 90$	H (1b)	0.50000	0.50000	0.50000
			Hf (1a)	0.00000	0.00000	0.00000
ZrH_{12}	$Pm\bar{3}$	$a = b = c = 3.0110$	H (12j)	0.20741	-0.00000	0.32468
		$\alpha = \gamma = \beta = 90$	Zr (1b)	0.50000	0.50000	0.50000
ZrH_{13}	$Pm\bar{3}$	$a = b = c = 3.0628$	H (12k)	0.50000	0.28446	0.84798
		$\alpha = \gamma = \beta = 90$	H (1b)	0.50000	0.50000	0.50000
			Zr (1a)	0.00000	0.00000	0.00000
LuH_{12}	$Pm\bar{3}$	a=b=c = 3.0093	H (12j)	0.21207	0.00000	0.33154
		$\alpha = \gamma = \beta = 90$	Lu (1b)	0.50000	0.50000	0.50000
LuH_{13}	$Pm\bar{3}$	$a = b = c = 3.0646$	H (12k)	0.50000	0.28435	0.85741
		$\alpha = \gamma = \beta = 90$	H (1b)	0.50000	0.50000	0.50000
			Lu (1a)	0.00000	0.00000	0.00000

Bader charges analysis

Table S2. Bader charges analysis of MH$_{12}$ and MH$_{13}$ at selected pressures. δ is the charge accepted on average by each H atom or lost on average by each M atom. H^1 and H^2 represent the H atoms in MH$_{13}$ occupying the six planes and the body center, respectively.

Structure	Pressure (GPa)	Atom	Charge value (e)	δ (e)
MgH_{12}	300	H	1.134	0.134
		Mg	6.392	1.608
MgH_{13}	300	H^1	1.116	0.116
		H^2	1.227	0.227
		Mg	6.381	1.619
ScH_{12}	300	H	1.095	0.095
		Sc	9.860	1.140
ScH_{13}	300	H^1	1.082	0.082
		H^2	1.192	0.192
		Sc	9.826	1.174
ZrH_{12}	300	H	1.116	0.116
		Zr	10.604	1.396
ZrH_{13}	300	H^1	1.110	0.110
		H^2	1.109	0.109
		Zr	10.572	1.428
HfH_{12}	300	H	1.119	0.119
		Hf	10.572	1.428
HfH_{13}	300	H^1	1.113	0.113
		H^2	1.116	0.116
		Hf	10.534	1.466
LuH_{12}	300	H	1.103	0.103
		Lu	23.760	1.240
LuH_{13}	300	H^1	1.091	0.091
		H^2	1.165	0.165
		Lu	23.739	1.261

Bond analysis

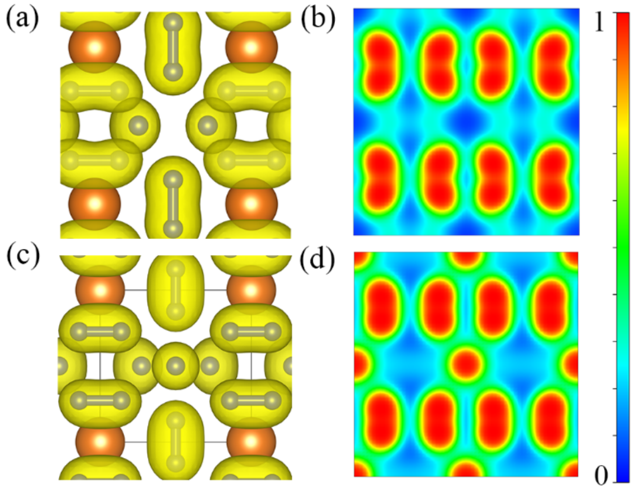

Figure S10. The calculated 3D ELF of (a) MgH_{12} and (c) MgH_{13} with isosurface value of 0.6, 2D ELF of (b) MgH_{12} and (d) MgH_{13} for the (0 0 2) plane at 300 GPa.

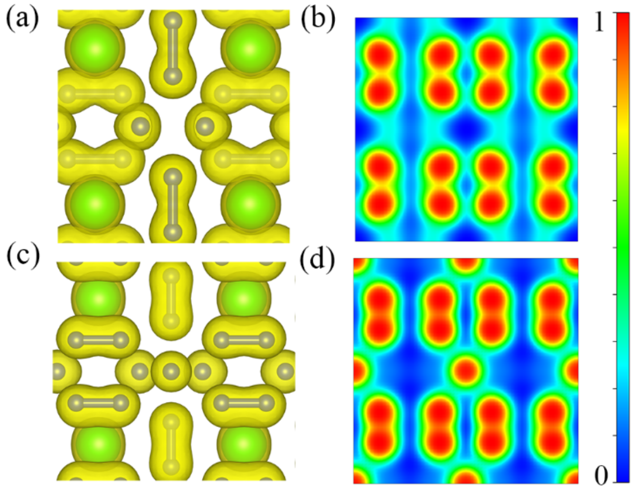

Figure S11. The calculated 3D ELF of (a) ZrH_{12} and (c) ZrH_{13} with isosurface value of 0.6, 2D ELF of (b) ZrH_{12} and (d) ZrH_{13} for the (0 0 2) plane at 300 GPa.

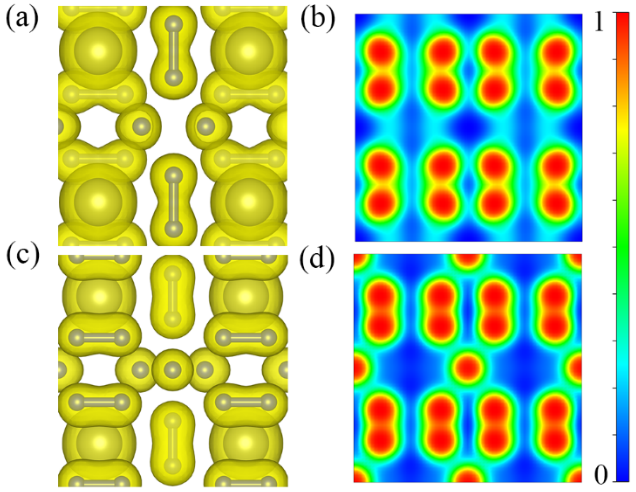

Figure S12. The calculated 3D ELF of (a) HfH_{12} and (c) HfH_{13} with isosurface value of 0.6, 2D ELF of (b) HfH_{12} and (d) HfH_{13} for the (0 0 2) plane at 300 GPa.

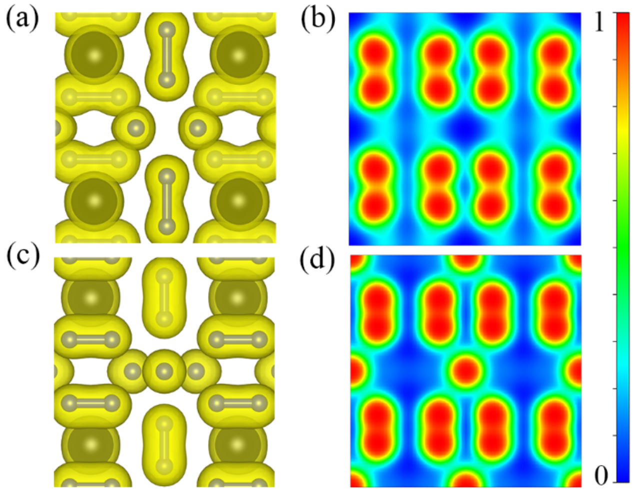

Figure S13. The calculated 3D ELF of (a) LuH_{12} and (c) LuH_{13} with isosurface value of 0.6, 2D ELF of (b) LuH_{12} and (d) LuH_{13} for the (0 0 2) plane at 300 GPa.

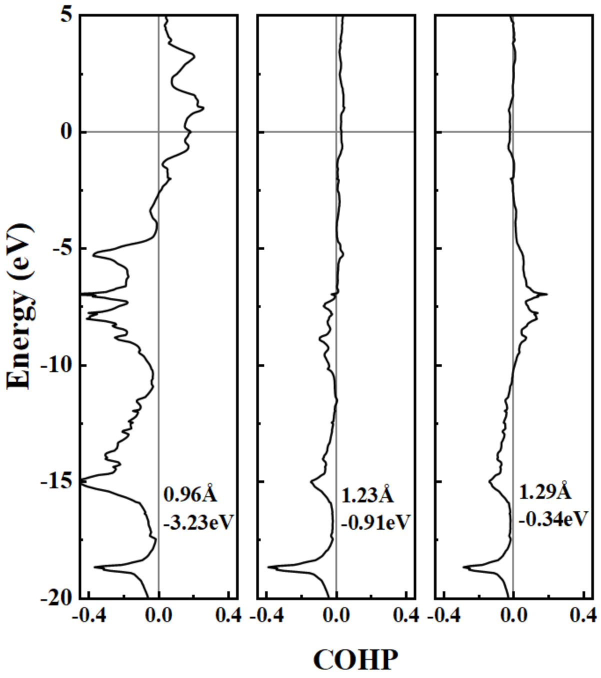

Figure S14. The calculated COHP of ScH_{12} 300 GPa. The corresponding H-H distances and values of ICOHP are labelled. The negative COHP indicates bonding, and the positive COHP indicates antibonding. The negative ICOHP values represent the bonding interactions between the H atoms.

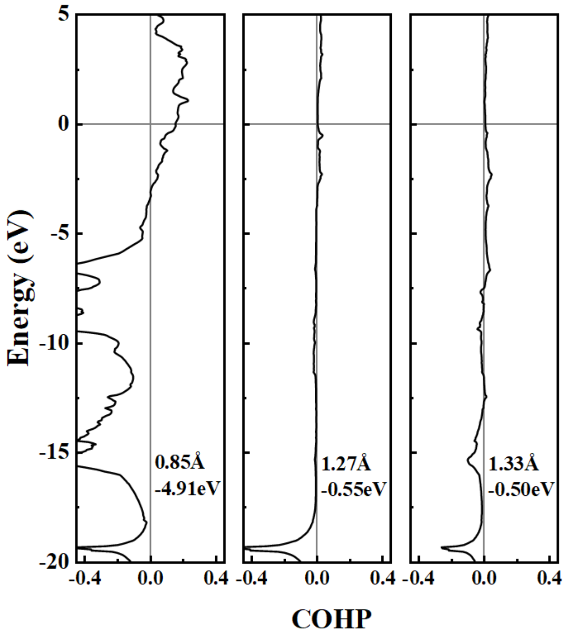

Figure S15. The calculated COHP of ScH_{13} at 300 GPa. The corresponding H-H distances and values of ICOHP are labelled.

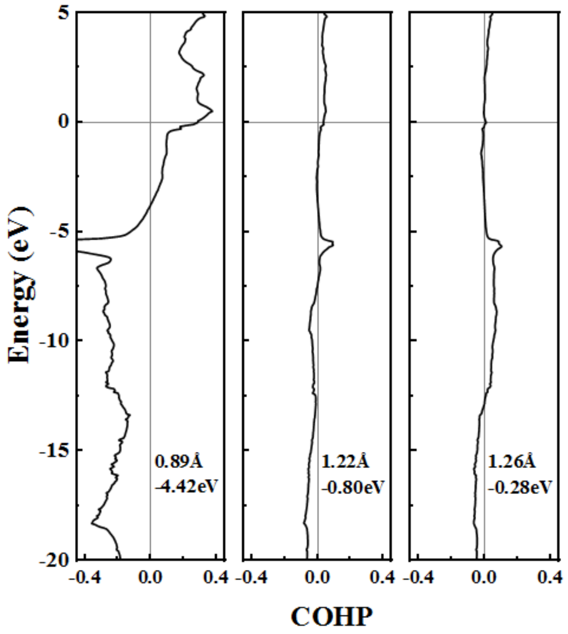

Figure S16. The calculated COHP of MgH_{12} at 300 GPa. The corresponding H-H distances and values of ICOHP are labelled.

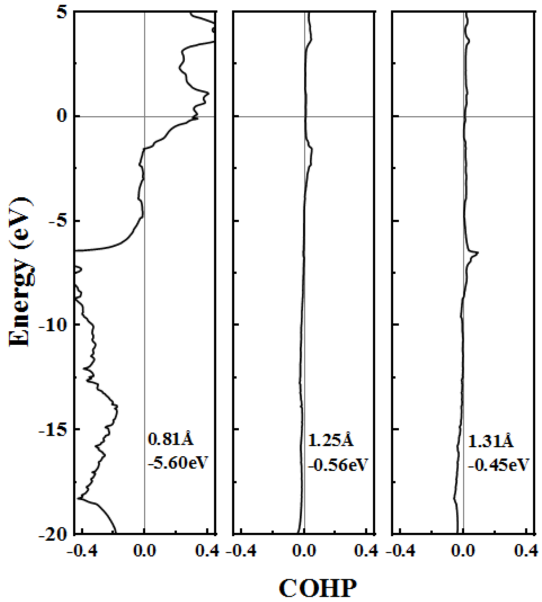

Figure S17. The calculated COHP of MgH_{13} at 300 GPa. The corresponding H-H distances and values of ICOHP are labelled.

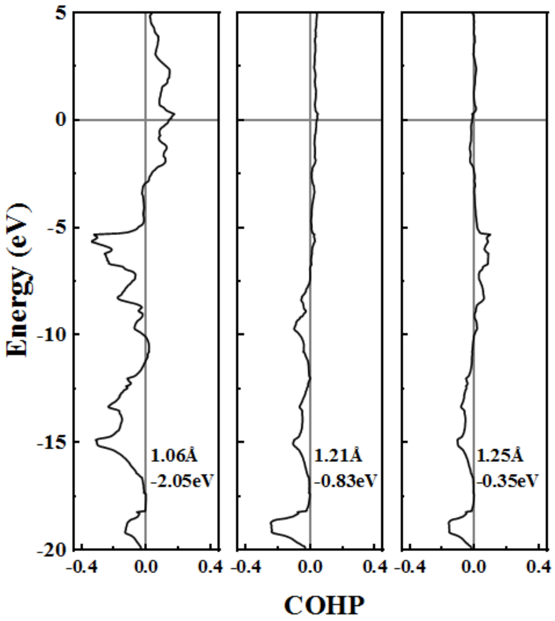

Figure S18. The calculated COHP of ZrH_{12} at 300 GPa. The corresponding H-H distances and values of ICOHP are labelled.

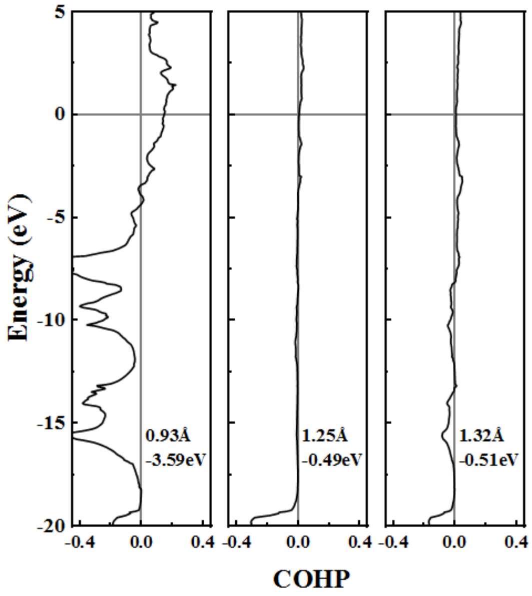

Figure S19. The calculated COHP of ZrH_{13} at 300 GPa. The corresponding H-H distances and values of ICOHP are labeled.

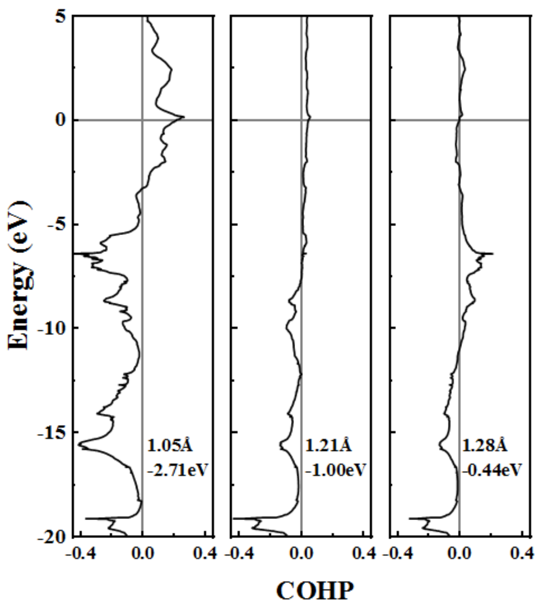

Figure S20. The calculated COHP of HfH_{12} at 300 GPa. The corresponding H-H distances and values of ICOHP are labeled.

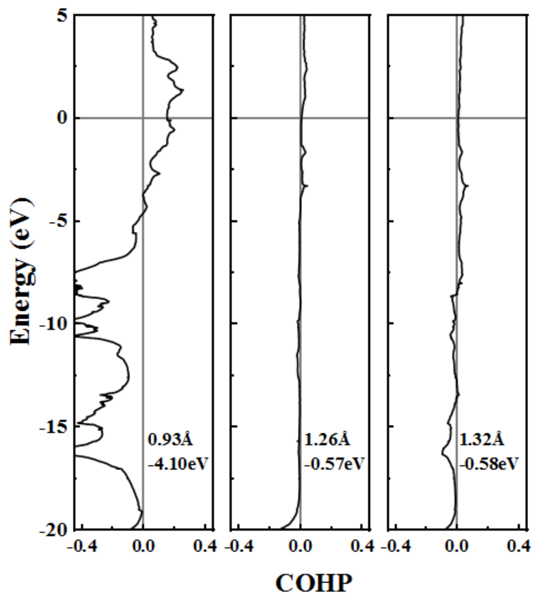

Figure S21. The calculated COHP of HfH_{13} at 300 GPa. The corresponding H-H distances and values of ICOHP are labelled.

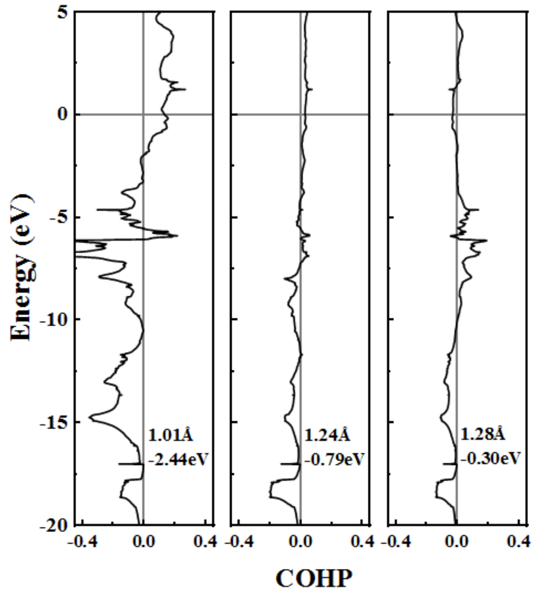

Figure S22. The calculated COHP of LuH_{12} at 300 GPa. The corresponding H-H distances and values of ICOHP are labeled.

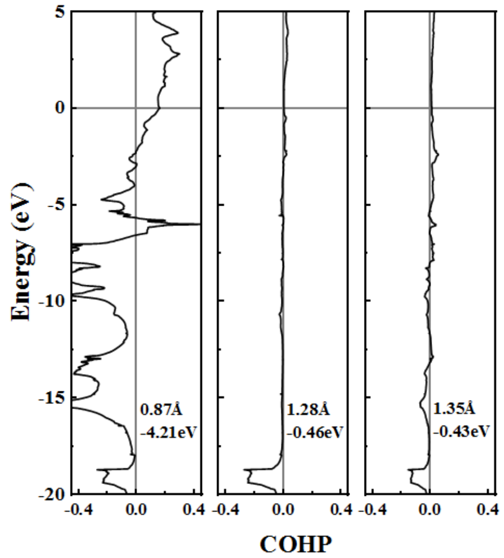

Figure S23. The calculated COHP of LuH_{13} at 300 GPa. The corresponding H-H distances and values of ICOHP are labeled.

Electronic properties analysis

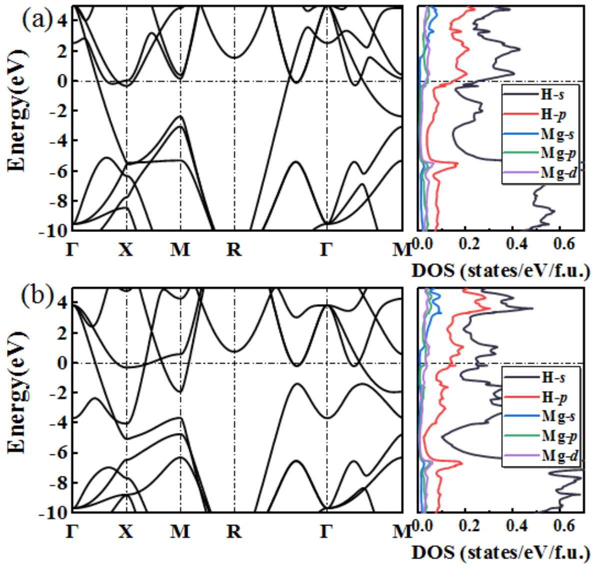

Figure S24. Calculated electronic band structure and partial DOS for (a) MgH_{12} and (b) MgH_{13} at 300 GPa.

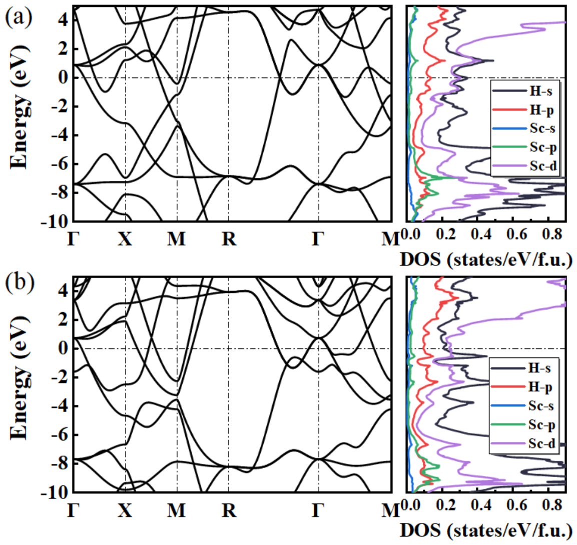

Figure S25. Calculated electronic band structure and partial DOS for (a) ScH_{12} and (b) ScH_{13} at 300 GPa.

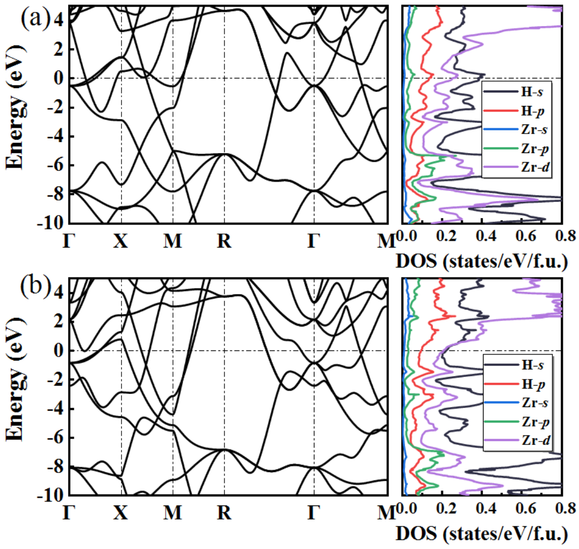

Figure S26. Calculated electronic band structure and partial DOS for (a) ZrH_{12} and (b) ZrH_{13} at 300 GPa.

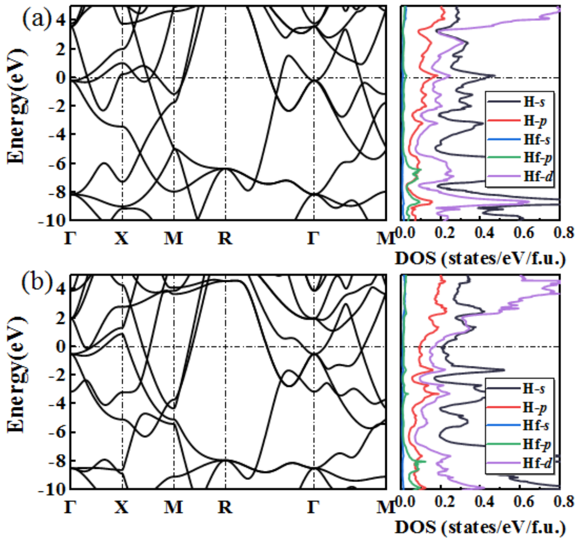

Figure S27. Calculated electronic band structure and partial DOS for (a) HfH_{12} and (b) HfH_{13} at 300 GPa.

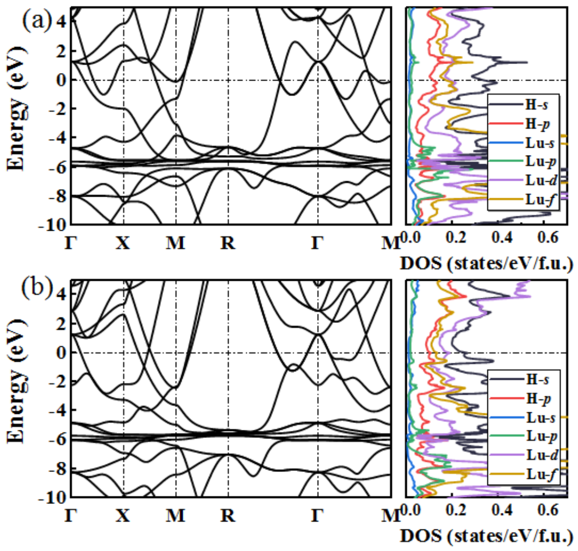

Figure S28. Calculated electronic band structure and partial DOS for (a) LuH_{12} and (b) LuH_{13} at 300 GPa.

Table S3. The calculated electronic DOS values of H and metal atoms in $MH_{12,13}$ at 300 GPa. The total DOS in this table is the sum of the DOS of H and metal atoms. The proportion of DOS of H is equal to the DOS of H atoms divided by the total DOS.

Structure	H atoms (states/eV/f.u.)	Metal atoms (states/eV/f.u.)	Total (states/eV/f.u.)	H proportion
MgH_{12}	0.38764	0.06734	0.45498	0.8520
MgH_{13}	0.37544	0.05819	0.43363	0.8658
ScH_{12}	0.47472	0.33205	0.80676	0.5884
ScH_{13}	0.30187	0.26548	0.56734	0.5321
ZrH_{12}	0.51358	0.30772	0.82131	0.6253
ZrH_{13}	0.31452	0.24649	0.56102	0.5606
HfH_{12}	0.57593	0.24414	0.82008	0.7023
HfH_{13}	0.28952	0.16981	0.45932	0.6303
LuH_{12}	0.44852	0.38565	0.83417	0.5377
LuH_{13}	0.36455	0.33912	0.70367	0.5181

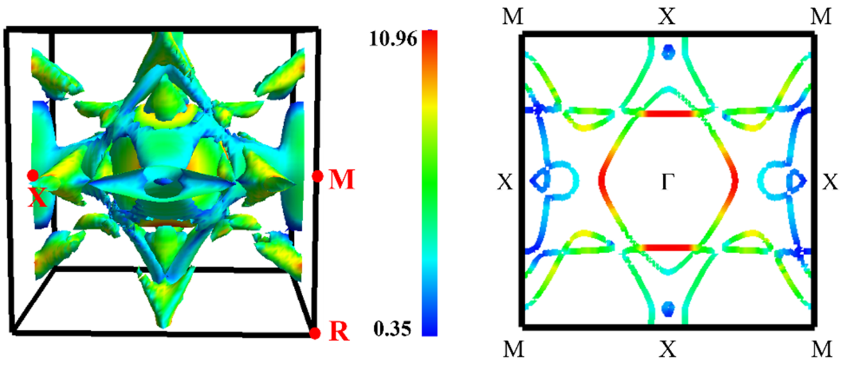

Figure S29. The Fermi surface of MgH_{12} at 300 GPa, with colors corresponding to different Fermi velocities (10^5 m/s).

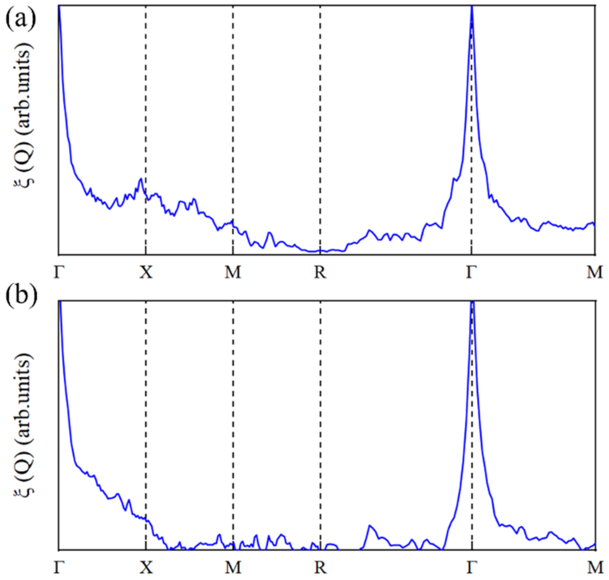

Figure S30. The calculated nesting function ξ (Q) of (a) MgH_{12} and (b) MgH_{13} at 300 GPa along some particular Q trajectories.

Phonon vibration modes

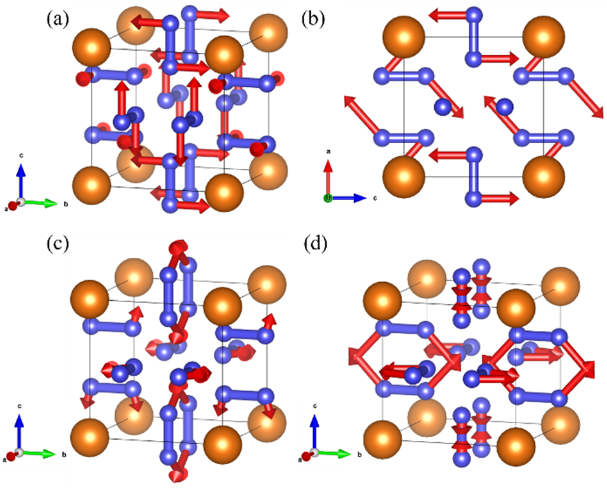

Figure S31. Visualization of the phonon vibration modes for strongest EPC at the Γ point in MgH_{12} at 300 GPa. (a) A_u mode at ~740 cm^{-1}, (b) T_g mode at ~850 cm^{-1}, (c) E_g mode at ~1740 cm^{-1}, and (d) E_{g*} mode at ~1740 cm^{-1}. The red arrows indicate the oscillation direction of the hydrogen ions.

Superconductive parameters

Table S4. The electron-phonon coupling parameter (λ), logarithmic average phonon frequency ($\omega_{\log}$), and superconducting transition temperature (T_c) of MH_{12} and MH_{13} with $Pm\bar{3}$ symmetry calculated using the Allen-Dynes-modified McMillan equation with correction factors ($f1f2 \neq 1$) and Eliashberg equations for $\mu^* = 0.1$-0.13.

Structure	Pressure (GPa)	λ	$\omega_{\log}$ (K)	N_{Ef} (States/spin/Ry/f.u.)	T_c (K) (μ^*=0.1-0.13) A-D-McMillan	T_c (K) (μ^*=0.1-0.13) Eliashberg
MgH_{12}	210	3.32	1213.6	5.33	300-326	356-376
	250	3.17	1279.3	5.20	305-331	360-385
	300	3.02	1339.7	5.06	307-334	366-388
	400	2.90	1352.2	4.81	304-331	375-398
ScH_{12}	90	4.24	744.4	8.67	235-257	297-313
	150	3.17	1046.7	7.27	250-272	299-320
	200	2.97	1131.4	6.73	256-279	306-325
	300	2.50	1322.6	6.10	258-281	304-325
ZrH_{12}	350	5.02	582.9	6.65	229-254	348-368
	400	3.57	908.9	6.39	252-276	335-360
HfH_{12}	360	6.08	406.0	6.64	197-223	367-388
	400	3.85	867.1	6.43	259-284	346-372
LuH_{12}	400	3.28	961.6	5.63	245-269	313-333
MgH_{13}	300	2.74	1116.3	4.58	242-265	303-324
	400	2.26	1401.4	4.29	252-276	301-324
ScH_{13}	90	1.81	807.2	5.93	117-129	135-148
	200	1.30	1322.7	4.86	127-144	138-154
	300	1.40	1404.3	4.80	149-167	166-185
ZrH_{13}	400	1.84	1017.4	4.77	151-167	186-204
HfH_{13}	450	2.30	559.4	4.45	103-114	192-211
LuH_{13}	100	1.73	925.9	5.29	128-141	150-164
	200	1.36	1183.6	4.52	122-137	137-154
	300	1.30	1312.5	4.41	127-143	143-161

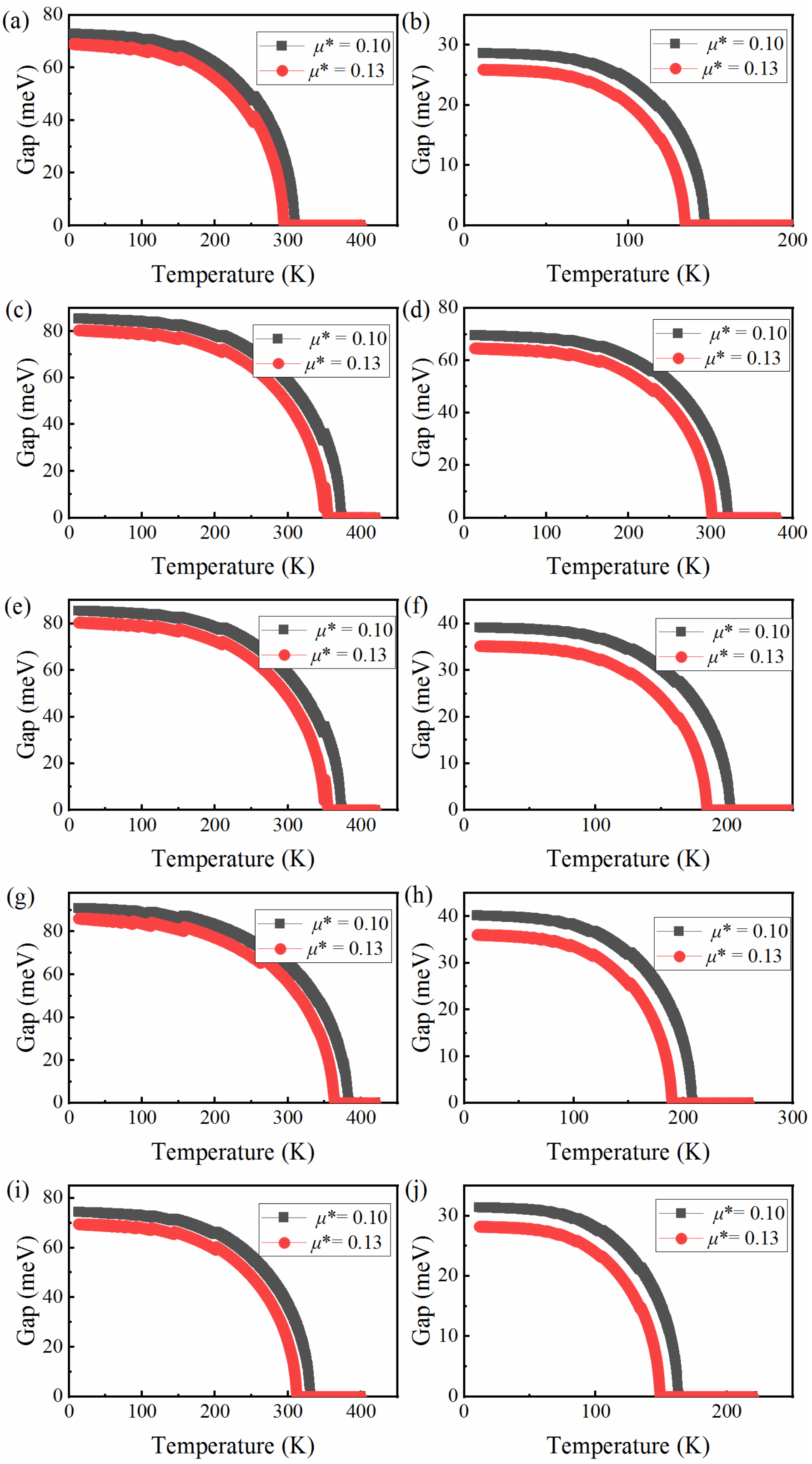

Figure S32. Calculated superconducting gap from Eliashberg equation of (a) ScH_{12} at 90 GPa (b) ScH_{13} at 90 GPa (c) MgH_{12} at 210 GPa (d) MgH_{13} at 300 GPa (e) ZrH_{12} at 350 GPa (f) ZrH_{13} at 400 GPa (g) HfH_{12} at 360 GPa (h) HfH_{13} at 450 GPa (i) LuH_{12} at 400 GPa (j) LuH_{13} at 100 GPa.

Anharmonic phonon correction and superconductivity

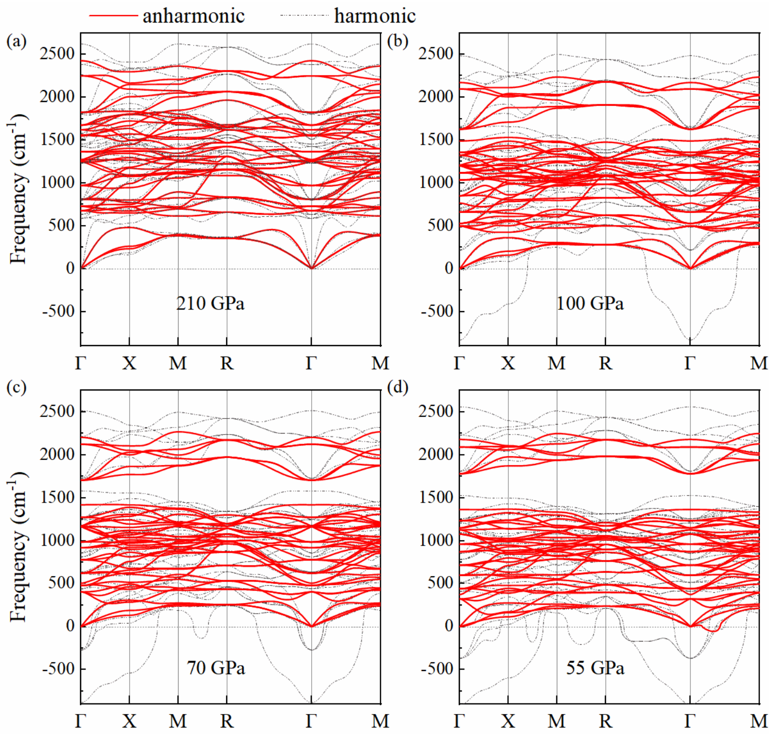

Figure S33. Comparison between the harmonic (red dash line) and anharmonic (red solid line) phonon band structure of MgH_{12} at (a) 210 GPa, (b) 100 GPa, (c) 70 GPa, and (d) 55 GPa.

Table S5. The electron-phonon coupling parameter (λ), logarithmic average phonon frequency (ω_{log}), and superconducting transition temperature (T_c) of MgH_{12} with $Pm\bar{3}$ symmetry calculated using the Allen-Dynes-modified McMillan equation and Eliashberg equations at the anharmonic level (^{Anh}) and the harmonic level (^{Har}).

Structure	Pressure (GPa)	λ	ω_{log} (K)	T_c (K) (μ*=0.1-0.13) A-D-McMillan	T_c (K) (μ*=0.1-0.13) Eliashberg
MgH_{12}	70^{Anh}	3.73	921.4	248-271	281-296
	100^{Anh}	3.36	1113.5	275-301	309-327
	210^{Anh}	2.94	1372.8	302-330	345-365
	210^{Har}	3.32	1213.6	300-326	356-376

Superconducting mechanism analysis

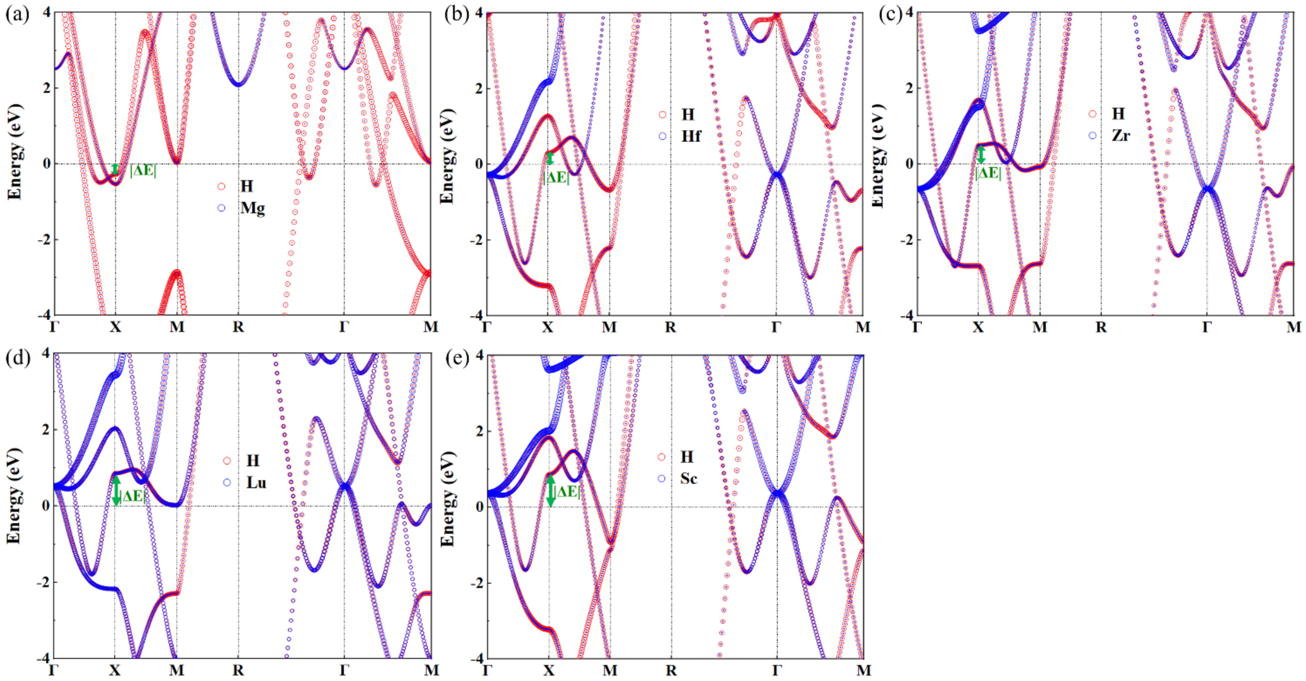

Figure S34. Electronic structure of MH12 at 400 GPa. The size of the red and blue circles represents the magnitude of the electronic DOS for H and M, respectively. The distance (|ΔE|) between the antibonding state of H-H and the Fermi energy level at the X point is labelled in the figure.

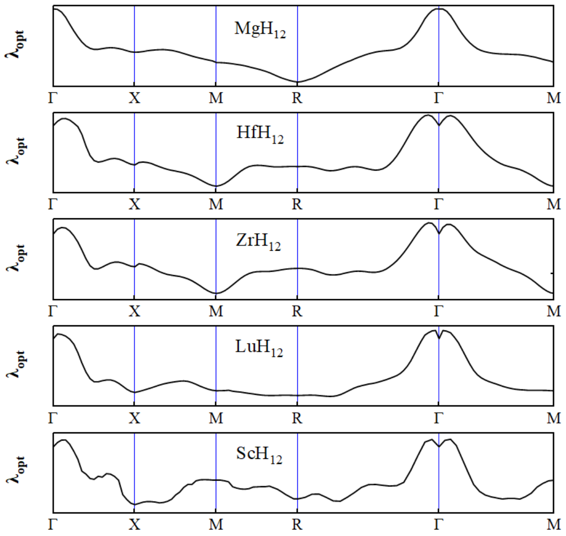

Figure S35. The contributions of the electron-phonon coupling constants of the optical modes (λ_{opt}) along high-symmetry paths in MH12 at 400 GPa.

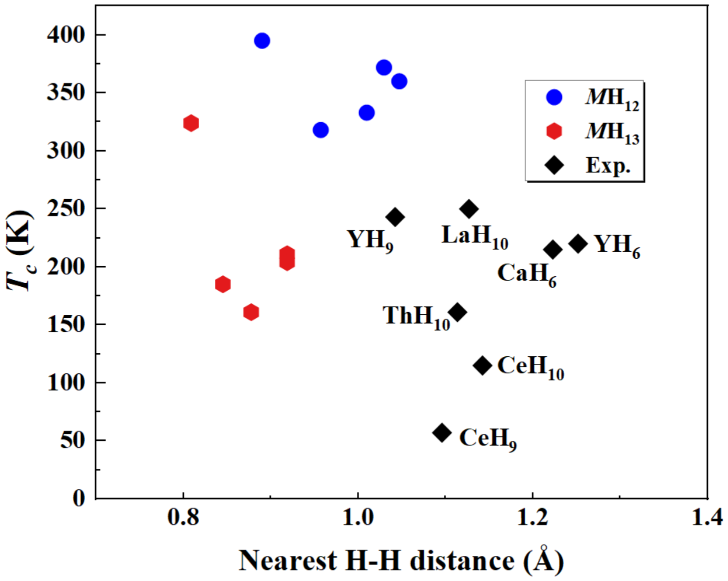

Figure S36. The comparison of T_c with the nearest H-H distance in hydrides.

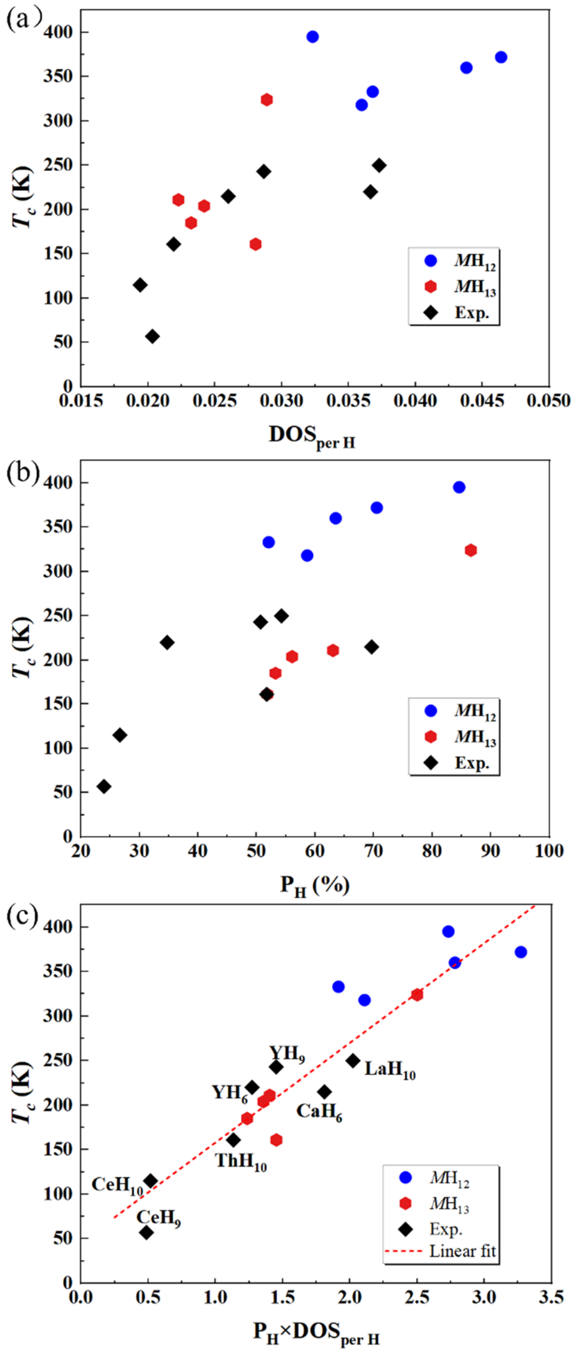

Figure S37. The comparison of T_c with (a) the percentage of the H electronic DOS at the Fermi level (P_H) and (b) the DOS per hydrogen atom ($DOS_{per\ H}$). (c) The proportion of hydrogen atoms in the total DOS at the Fermi energy multiplied by the DOS per hydrogen atom, plotted against T_c values.

References

[1] C. J. Pickard, R. J. Needs, *Phys. Rev. Lett.* **2006**, *97*, 045504.

[2] C. J. Pickard, R. J. Needs, *J. Phys.: Condens.Matter* **2011**, *23*, 053201.

[3] S. J. Clark, M. D. Segall, C. J. Pickard, P. J. Hasnip, M. I. J. Probert, K. Refson, M. C. Payne, *Zeitschrift für Kristallographie - Crystalline Materials* **2005**, *220*, 567.

[4] P. Hohenberg, W. Kohn, *Physical Review* **1964**, *136*, B864.

[5] W. Kohn, L. J. Sham, *Physical Review* **1965**, *140*, A1133.

[6] H. J. Monkhorst, J. D. Pack, *Phys. Rev. B* **1976**, *13*, 5188.

[7] J. P. Perdew, K. Burke, M. Ernzerhof, *Phys. Rev. Lett.* **1996**, *77*, 3865.

[8] G. Kresse, J. Furthmuller, *Phys Rev B Condens Matter* **1996**, *54*, 11169.

[9] P. E. Blochl, *Phys Rev B Condens Matter* **1994**, *50*, 17953.

[10] A. D. Becke, K. E. Edgecombe, *The Journal of Chemical Physics* **1990**, *92*, 5397.

[11] R. Dronskowski, P. E. Bloechl, *The Journal of Physical Chemistry* **1993**, *97*, 8617.

[12] R. F. W. Bader, *Acc. Chem. Res.* **1985**, *18*, 9.

[13]

[14] A. Togo, F. Oba, I. Tanaka, *Phys. Rev. B* **2008**, *78*, 134106.

[15] P. Giannozzi, S. Baroni, N. Bonini, M. Calandra, R. Car, C. Cavazzoni, D. Ceresoli, G. L. Chiarotti, M. Cococcioni, I. Dabo, A. Dal Corso, S. de Gironcoli, S. Fabris, G. Fratesi, R. Gebauer, U. Gerstmann, C. Gougoussis, A. Kokalj, M. Lazzeri, L. Martin-Samos, N. Marzari, F. Mauri, R. Mazzarello, S. Paolini, A. Pasquarello, L. Paulatto, C. Sbraccia, S. Scandolo, G. Sclauzero, A. P. Seitsonen, A. Smogunov, P. Umari, R. M. Wentzcovitch, *J. Phys.: Condens.Matter* **2009**, *21*, 395502.

[16] K. F. Garrity, J. W. Bennett, K. M. Rabe, D. Vanderbilt, *Computational Materials Science* **2014**, *81*, 446.

[17] P. B. Allen, R. C. Dynes, *Physical Review B* **1975**, *12*, 905.

[18] G. M. Eliashberg, *Sov. Phys. - JETP* **1960**, *11*, 696.

[19] R. Bianco, I. Errea, L. Paulatto, M. Calandra, F. Mauri, *Phys. Rev. B* **2017**, *96*.

[20] L. Monacelli, I. Errea, M. Calandra, F. Mauri, *Phys. Rev. B* **2018**, *98*.
